# Metformin directly suppresses atherosclerosis in normoglycaemic mice via haematopoietic adenosine monophosphate-activated protein kinase

**DOI:** 10.1093/cvr/cvaa171

**Published:** 2020-06-25

**Authors:** Anusha Seneviratne, Luke Cave, Gareth Hyde, Soren Kragh Moestrup, David Carling, Justin C Mason, Dorian O Haskard, Joseph James Boyle

**Affiliations:** 1 National Heart and Lung Institute, Imperial College London, London, UK; 2 Department of Biomedicine, Aarhus University, Aarhus C, Denmark; 3 MRC London Institute of Medical Sciences, Imperial College London, UK

**Keywords:** Atherosclerosis, Macrophage, Metformin, AMPK, Gene expression, Transcription factor

## Abstract

**Aims:**

Atherosclerotic vascular disease has an inflammatory pathogenesis. Heme from intraplaque haemorrhage may drive a protective and pro-resolving macrophage M2-like phenotype, Mhem, via AMPK and activating transcription factor 1 (ATF1). The antidiabetic drug metformin may also activate AMPK-dependent signalling. *Hypothesis:* Metformin systematically induces atheroprotective genes in macrophages via AMPK and ATF1, thereby suppresses atherogenesis.

**Methods and results:**

Normoglycaemic *Ldlr*^−/−^ hyperlipidaemic mice were treated with oral metformin, which profoundly suppressed atherosclerotic lesion development (*P* < 5 × 10^−11^). Bone marrow transplantation from AMPK-deficient mice demonstrated that metformin-related atheroprotection required haematopoietic AMPK [analysis of variance (ANOVA), *P* < 0.03]. Metformin at a clinically relevant concentration (10 μM) evoked AMPK-dependent and ATF1-dependent increases in *Hmox1*, *Nr1h2* (*Lxrb*), *Abca1*, *Apoe*, *Igf1*, and *Pdgf*, increases in several M2-markers and decreases in *Nos2*, in murine bone marrow macrophages. Similar effects were seen in human blood-derived macrophages, in which metformin-induced protective genes and M2-like genes, suppressible by si-ATF1-mediated knockdown. Microarray analysis comparing metformin with heme in human macrophages indicated that the transcriptomic effects of metformin were related to those of heme, but not identical. Metformin-induced lesional macrophage expression of p-AMPK, p-ATF1, and downstream M2-like protective effects.

**Conclusion:**

Metformin activates a conserved AMPK-ATF1-M2-like pathway in mouse and human macrophages, and results in highly suppressed atherogenesis in hyperlipidaemic mice via haematopoietic AMPK.

## 1. Introduction

Atherosclerotic cardiovascular disease is a major cause of morbidity and mortality in developed nations.^1^ It is therefore still an area of unmet therapeutic need. Atherosclerosis comprises chronic inflammation of the intima of medium-sized and large arteries, in which the inflammatory cells are primarily macrophages.^2^ This leads to major adverse cardiovascular events (MACE): ischaemia of the heart, brain, or periphery.^1^ Inflammation is a major pathological driver of atherosclerosis.

The risk factors for atherosclerosis include hypertension, hyperlipidaemia, diabetes, smoking, and genetics.^1^ The increasing prevalence and severity of obesity has made Type 2 diabetes mellitus (T2DM) an important cause of cardiovascular disease.^1^ The current pharmacological first-line treatment of T2DM is metformin, an insulin-sensitizing hypoglycaemic agent that has proven very safe in 50 years of clinical practice. It was once thought that glucose-reduction itself would decrease inflammatory stimulation and atherosclerosis.^3^^,^^4^ Importantly, in several randomized controlled trials, metformin had a possible effect on diabetic macrovascular disease not seen with other oral hypoglycaemic agents.^5–9^ This raises the possibility of a mechanism additional to glucose-reduction. If correct, it would indicate that metformin might be ‘repurposed’ for direct vascular risk reduction.

Multiple studies have indicated several possible molecular mechanisms of action of metformin. Metformin may act via inhibition of mitochondrial electron transport chain Complex-I,^10^ or via inhibition of the mitochondrial enzyme GPD2,^11^ or possibly by modulating glucagon-mediated activation of adenylate cyclase.^12^ There is clear evidence that metformin activates AMPK.^13^ However, not all of the effects of metformin are AMPK-mediated. Indeed, in the highly aggressive streptozotocin-diabetes model *ApoE*^−/−^ model of profound Type I diabetes and hyperlipidaemia, metformin acted via the methylglyoxal pathway to reduce atherosclerosis, and independently of AMPK.^14^ Importantly, Viollet’s group have recently shown that its key therapeutic effect, reducing blood glucose by acting on the liver, is not AMPK-dependent.^13^ Therefore, the role of AMPK in metformin responses *in vivo* is currently uncertain.

Metformin has multiple AMPK-mediated beneficial effects on vascular physiology and normal vessel wall cells [endothelial cells and vascular smooth muscle cells (VSMCs)].^15^ Metformin has multiple other potential effects, including modulation of macrophage function.^16^^,^^17^ In these studies, it acted effectively as an anti-inflammatory agent.^16^^,^^17^ Our interest stemmed from analysing the endogenous activating transcription factor 1 (ATF1)-dependent homeostatic macrophage phenotype, Mhem.^18^ This phenotype was driven by intraplaque haemorrhage.^19^^,^^20^ Whilst Mhem is different to M2, it is nevertheless conceptually M2-like in that it is homeostatic.^18^ We showed that metformin-modulated HO-1, oxidative stress, and lipid handling in human blood-derived macrophages, mediated by AMPK.^17^ Genetically mediated AMPK activation suppresses macrophage activation.^21^

We postulated a direct mechanism mediated by plaque macrophage AMPK, similar to the one that we characterized *in vitro.*^17^ This was tested in chow-fed *Ldlr*^−/−^ mice, an *in vivo* atherosclerosis model with enhanced clinical relevance that we have published.^21–26^ These develop small early lesions limited to the thoracic aorta and composed predominantly of macrophages.^22–26^ Metformin profoundly suppressed lesion formation in *Ldlr*^−/−^ mice dependent on haematopoeitic AMPK. Metformin-induced M2-like macrophage genes, along with multiple other atheroprotective genes via AMPK and ATF1 in humans and mice. This may have therapeutic relevance for the repurposing of metformin by utilizing its so-called pleiotropic effects.^27^

## 2. Methods

Methods are detailed in the Supplementary material online. *Ldlr*^−/−^ mice were studied in experiments that were designed to give complementary information. Chow was used throughout similar to our previous studies,^21–26^ since high-fat diet is associated with dysglycaemia and would have confounded assessment of glucose and diabetes-associated inflammation. The standard age at analysis was 25 weeks, which on chow produces early atherosclerosis. Lesion assessment, histology, immunohistology, cell culture of mouse bone marrow-derived macrophages (mBMMs), and human monocyte-derived macrophages (hMDMs), and reverse transcription and polymerase chain reaction (RT-qPCR) were all by standard methods (Supplementary material online). Microarrays and bioinformatics followed methods in our previous study.^18^

In brief, mice were maintained on metformin (Sigma-Aldrich D150959-5G) in drinking water (1 mg⋅mL^−1^) for the study duration (Supplementary material online). Saccharin was added to disguise the taste. Animals were euthanized, perfusion-fixed, the aortae and aortic roots removed. Aortic roots were cryosectioned and stained with Oil Red O. Aortae were stained with Sudan-IC during perfusion, dissected, opened out and imaged. Immunohistology was carried out on spare aortic root cryosections using immunoperoxidase, immunofluorescence, and immunoalkaline phosphatase protocols for cell signalling molecules and cell lineage markers. Mouse bone marrow-derived macrophages were cultured in L929-conditioned medium and transferred to 24-cell plates for stimulation (Supplementary material online). The macrophages were lysed and RNA purified with a Qiagen RNEasy Mini kit (Supplementary material online). RNA was reverse transcribed and measured with qPCR using a SyBr Green-based mastermix (Supplementary material online).

Human monocyte-derived macrophages were obtained from blood aseptically collected from normal human volunteers (HTA Licence 12275 subcollection VAS_JB-17-076), and monocytes purified using a Ficoll-Hypaque density layer and adherence-purification. Human peripheral blood was obtained with informed consent and favourable ethical opinion from Hammersmith Research Ethics Committee. All procedures conformed to the principles outlined in the Declaration of Helsinki. Monocytes were cultured overnight before stimulation with metformin. Metformin was 10 μmol⋅L^−1^ final concentration. RNA purification and RT-qPCR was as for the mouse macrophages, but using an RNEasy Micro kit and human-specific primers (Supplementary material online). Metformin-stimulated macrophages were lysed, RNA purified and labelled and hybridized to an Agilent 4x44k microarray system. Genespring, Venn analysis, X2K (https://amp.pharm.mssm.edu/X2K/), PANTHER-GO (http://www.pantherdb.org/), and STRING (https://string-db.org/) (Supplementary material online).

The *in vivo* experiments had local approval (Local AWERB and UK Home Office PC54F329C and 70/7325) and all procedures conformed to the guidelines from Directive 2010/63/EU of the European Parliament on the protection of animals used for scientific purposes. Euthanasia was by excess CO_2_ over 7 min with confirmation of death by cervical dislocation. No anaesthetic agents were required.

Statistics were done in SigmaStat (SigmaPlot). Data are shown as scatterplots where informative or as mean ± standard error. All data were tested for normality by Shapiro–Wilk. Normally distributed data in two groups were tested with Student’s *t*-test, and non-normally distributed data in two groups by Wilcoxon–Mann–Whitney. Normally distributed data in multiple groups were tested by one-way analysis of variance (ANOVA) with Bonferroni’s correction. Non-normally distributed data were tested by ANOVA with Holm–Sidak post-testing. Paired data were tested using the paired versions of *t*-Student or Wilcoxon.

## 3. Results

### 3.1 Metformin reduces atherosclerosis without affecting glucose or lipids

**Figure 1:**
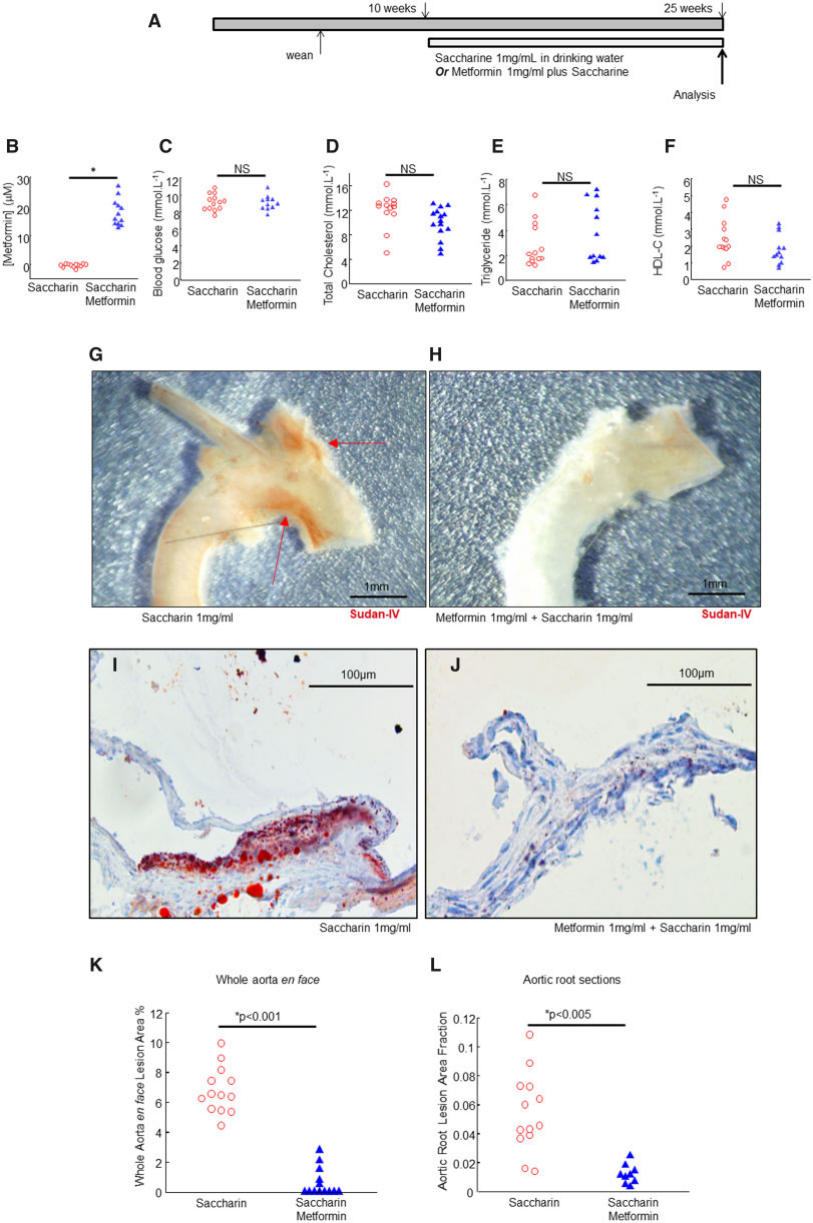
Metformin does not modulate blood glucose or lipids but profoundly reduces early atherosclerotic lesions. (*A*) Graphical outline of experimental protocol. Ldlr−/− mice were fed a chow diet, with metformin or saccharin in drinking water from 10 weeks and analysed at 25 weeks. (*B*–*F*) Serum analytes as indicated, in metformin-treated or control mice as indicated. *P < 0.05, Student’s *t*-test used where indicated. The glucose measurements were random (non-fasted) on a drop of blood from the tail vein the day before sacrifice, using an Aviva Nano device and strips. (*G* and *H*) Representative magnified images of aortic arches, as indicated, scalebars, 1 mm as indicated. (*I* and *J*) Representative images of aortic roots, as indicated, stained with Oil-Red O and haematoxylin, scalebars 100 μm as indicated. (*K* and *L*) Statistical analysis of lesions in aortic root and aorta, based on imaging in *I*, *J*. *K*, *Y*-axis, aortic en face, *n* = 13 mice (saccharin) *n* = 13 mice (metformin), *significant, indicated *P*-value, Wilcoxon–Mann–Whitney. (*L*) Aortic root, *n* = 13 mice (saccharin) *n* = 9 mice (metformin) *significant, indicated *P*-value, Wilcoxon–Mann–Whitney.

In the first set of experiments, we began *Ldlr*^−/−^ chow-fed mice on oral metformin at age 10 weeks, culling for analysis at 25 weeks (*Figure 1A*). The model and timings were based on our previous studies.^22–26^ Metformin was administered under published conditions at an oral dose that approximated clinical concentrations in humans.^28^ Metformin was administered in drinking water (1 mg⋅mL^−1^) with saccharin (1 mg⋅mL^−1^) to disguise its taste, or matched saccharin-only controls in drinking water (1 mg⋅mL^−1^).^29^ Metformin administration yielded detectable metformin in serum but did not detectably modulate body weight, near-terminal random blood glucose, low-density lipoprotein cholesterol, high-density lipoprotein cholesterol, or triglycerides (*Figure 1B*–*F* and not shown).

Murine atherosclerosis was quantified using standard methods, with lipid stains in the aorta and aortic root (*Figure 1G*). Consistent with our previous reports,^22–26^ this very mild model of atherosclerosis (chow-fed *Ldlr*^−/−^) had early lesions around the inner arch and major branch-points of the arch with sparing of the descending aorta (*Figure 1G* and Supplementary material online, *Figure SI*). Lesion development was profoundly inhibited by metformin administration (*Figure 1H*). Similarly, the aortic roots had small early lesions comprising collections of foamy macrophages at the valve bases (*Figure 1I*). Lesion development at this site was also profoundly inhibited by metformin (*Figure 1J*). The aorta *en face* lesion area was calculated along the whole aorta and the aortic root lesion area was calculated from sections as before.^22–24^ Statistically, this effect was consistent when measured at both sites (*Figure**1**K* and *L*).

Next, lesion composition was assessed. The lesions in the saccharin controls corresponded to those expected of this age and diet in *Ldlr*^−/−^, being entirely composed of macrophages (CD68+) (Supplementary material online, *Figure SIA*–*D*), without significant lesional collagen or VSMCs. The very few lesions that did occur in the treated mice, had a composition similar to those in the untreated. The strength of suppression of lesions precluded further analysis.

### 3.2 Atherosclerosis-prevention by metformin requires haematopoietic AMPK

**Figure 2:**
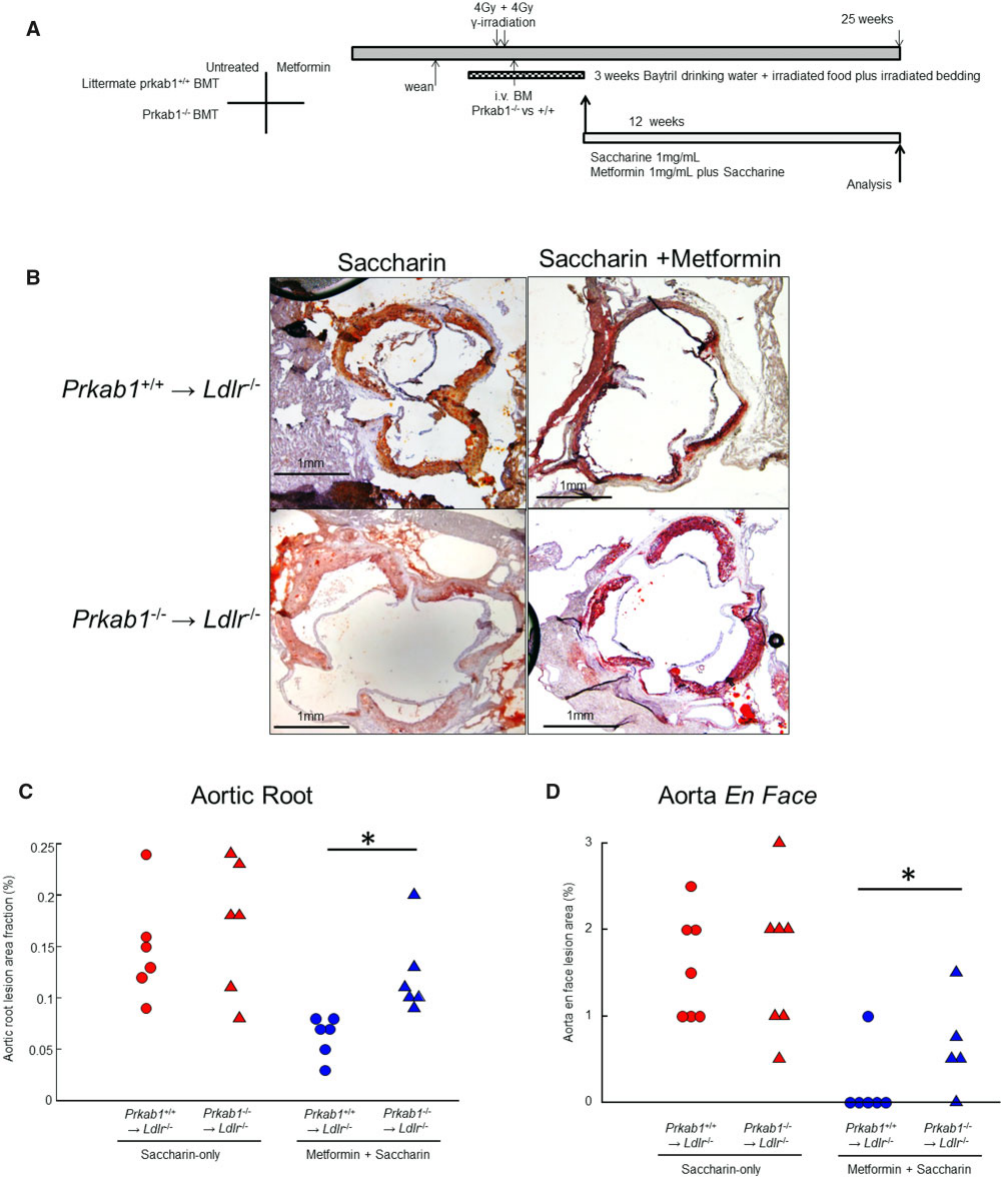
Metformin reduces early development of atherosclerosis via haematopoietic AMPK. (*A*) Graphical outline of experimental protocol. Mice were culled for analysis at 25 weeks to match the first cohort and had BMT at approximately 12 weeks age. (*B*) Representative images of Ldlr−/− mice with *Prkab*^+/+^ or *Prkab*1^−/−^ BMT that were fed a chow diet with metformin or saccharin, as indicated, with Oil-Red-O staining and imaging as in *Figure 1*. *Ldlr*^−/−^ chow 25 weeks (*Prkab*1^+/+^→*Ldlr*^−/−^saccharin, *n* = 6, *Prkab*1^−/−^→*Ldlr*^−/−^saccharin, *n* = 6, *Prkab*1^+/+^→*Ldlr*^−/−^metformin, *n* = 6, *Prkab*1^−/−^→*Ldlr*^−/−^metformin, *n* = 6), scalebars 1 mm as indicated, and quantified in *Figure 2C*. (*C*) Statistical analysis of lesion size in aortic roots based on imaging in B (Oil-Red-O staining). *P < 0.03, ANOVA, with Holm–Sidak adjustment, after normality testing (Shapiro–Wilk), *n* = 6 mice, each point represents a different mouse. (*D*) Statistical analysis of lesion size in aortae en face (*n* = 5–7 mice, each point represents a different mouse, Sudan-IV staining, imaging as in *Figure 1*). *P < 0.05, Student’s *t*-test for indicated comparison, after normality testing (Shapiro–Wilk).

We next asked whether this effect was mediated by haematopoietic AMPK, with a bone marrow transplantation (BMT) approach (*Figure 2*). This approach is standard in the atherosclerosis literature and is usually interpreted as indicating myeloid specificity and even implied specificity for macrophages.^30–34^ Recipient *Ldlr*^−/−^ mice were preconditioned with lethal γ-irradiation, then rescued by adoptive transfer of bone marrow from mice deficient in AMPK (*Prkab1*^−/−^) or littermate matched (*Prkab1*^+/+^) controls. BMT was performed at age 12 weeks, and metformin treatment from age 14 weeks to age 25 weeks, allowing a prolonged period on metformin after BMT, when there was a cull for analysis as for the first cohort (*Figure 2A*). Over 90% of lesional cells were negative for immunostainable PRKAB1 in the mice reconstituted with *Prkab1*^−/−^ marrow (Supplementary material online, *Figure SIF*). On genotype analysis of peripheral blood by qPCR, there was no difference in reconstitution between saccharin and metformin (Supplementary material online, *Figure SIG*).

Neither BMT nor metformin had a significant effect on weight (Supplementary material online, *Figure SII*). Baseline lesion development at the aortic root was slightly higher than the first cohort, possibly due to irradiation (*Figure*2*B and C*). Metformin again profoundly suppressed lesion development although not quite to zero. Importantly, the effect of metformin was lost in mice that had been transplanted from AMPK-deficient donors. The lesions were entirely composed of macrophages (CD68+) (Supplementary material online, *Figure SIA*–*D*), without significant lesional collagen or VSMCs (not shown). This was similar to the non-irradiated controls and to the composition in *Ldlr*^−/−^ on chow at this age that we and others have previously reported.^22–26^ In aorta *en face* analysis, again metformin suppressed lesion development, and this effect was also prevented by BMT from *Prkab1*^−/−^ mice (*Figure 2D*). Reconstitution was analysed by both peripheral blood qPCR and by immunostaining of the lesions for PRKAB1 protein. We found that atherosclerotic lesions did not contain PRKAB1 in mice reconstituted with *Prkab1*^−/−^ marrow (Supplementary material online, *Figure SIF*). Moreover, there was no difference in reconstitution between metformin-treated and control mice (Supplementary material online, *Figure SIG*). This experiment indicated that the anti-atherosclerotic effects of metformin were mediated by haematopoietic cell AMPK.

### 3.3 Metformin induces macrophage atheroprotective genes via AMPK and ATF1

**Figure 3:**
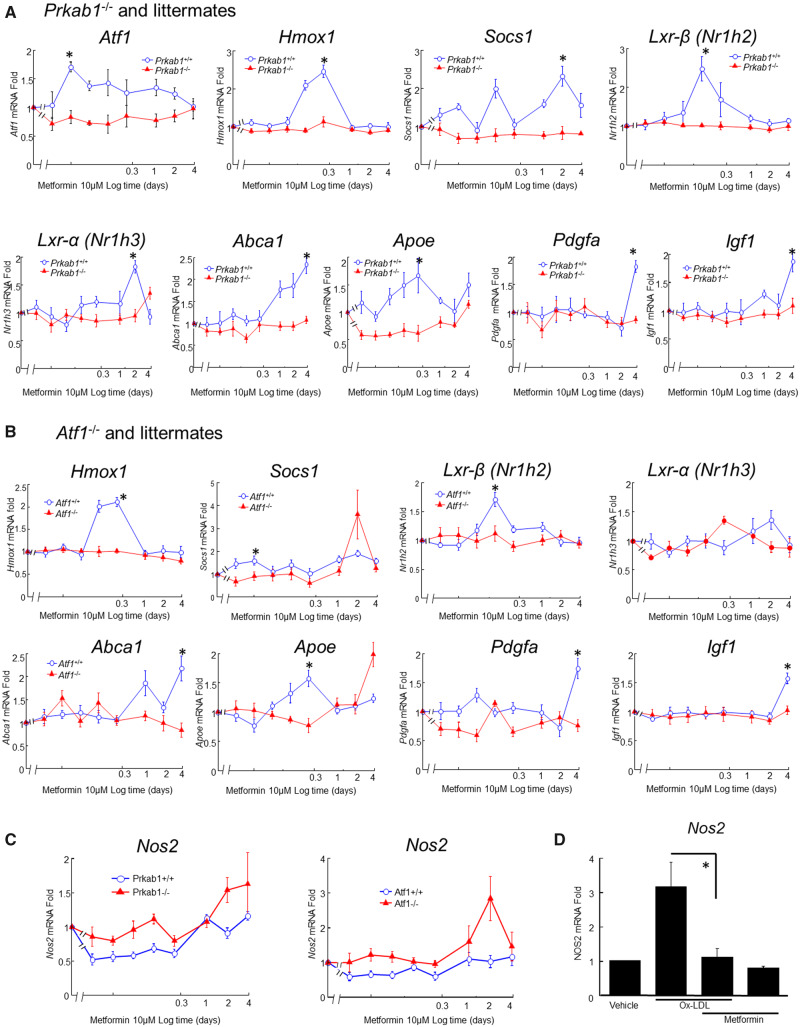
Metformin induces atheroprotective gene in mBMM dependent on AMPK and ATF1. Genes are as indicated on each sub-graph. *X*-axes, log time scale after stimulation of mBMM (in days). *Y*-axes, fold induction from untreated controls (calculated by −ΔΔCt method). Modulated genes, knockouts and littermate controls are as indicated, either *Prkab*1^−/−^ (*A*) or *Atf1*^−/−^ (*B*). In *C*, repression of iNOS (Nos2) is shown under baseline conditions. Metformin represses expression of Nos2, and this repression is dependent on both *Prkab*1 and Atf1. Statistical testing was by ANOVA with Holm–Sidak *post hoc* correction, after normality testing (Shapiro–Wilk), *n* = 6–8 different mice per condition. (*D*) In hMDM, metformin repression of OxLDL-mediated induction of Nos2 is clearer when OxLDL is used to induce NOS2, the repression is clearer. Mean ± SE, *P < 0.05, *n* = 5 different mice.

To identify possible AMPK-dependent molecular mechanisms, mBMMs were studied using RT-qPCR to quantify gene activation. Responses were followed over a time-course to identify optimal time points for dynamic responses.^18^ The gene selection was hypothesis-driven, based on previous work^18^ and is summarized in Supplementary material online, *Figure SIII*. Metformin-induced *Atf1*, *Hmox1*, *Socs1*, *Lxra*, *Lxrb*, *Abca1*, *Apoe*, *Pdgf*, and *Igf1* (*Figure 3*). These genes were induced in a dynamic pattern, with each gene peaking at different time points (*Figure*3*A and B*).

Most of the gene induction was lost in mBMM from mice deficient in either *Prkab1* or *Atf1* (*Figure*3*A and B*). That is, both AMPK and ATF1 were required for these responses to metformin. These data therefore indicated that the majority of the atheroprotective effects of metformin on macrophages were mediated by *Atf1*. This is likely to be due to AMPK activating ATF1, as previously characterized.^17^ Study of *Prkab1*^−^^*/*^^−^ x *Atf1*^−^^*/*^^−^ double-knockouts was precluded by loss during pregnancy (not shown). *Nos2* was also assessed, as it has been previously linked to macrophage activation by oxidized low-density lipoproteins (OxLDLs)^35^^,^^36^ and to atherogenesis.^37–40^ Metformin suppressed *Nos2* expression, both basally in mBMM and in hMDM when induced by adding OxLDL (*Figure*3*C and D*).

**Figure 4:**
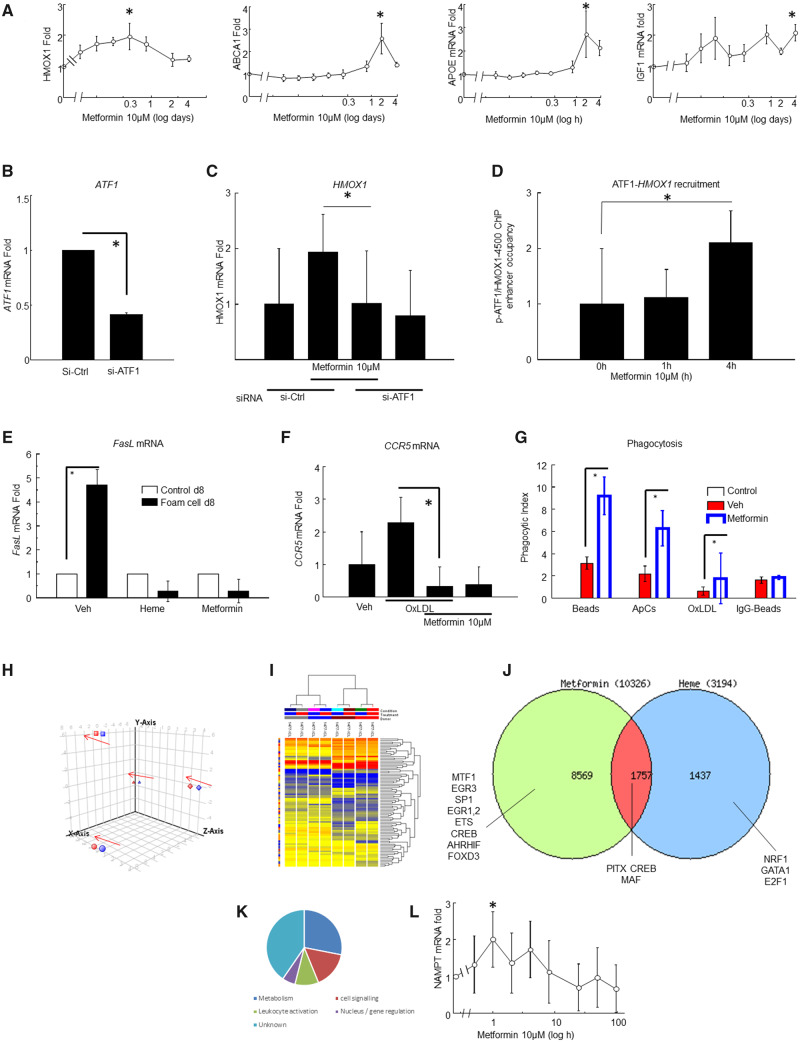
Metformin induces Mhem-like atheroprotective genes in hMDM. (*A*) Metformin induces HMOX1, APOE, ABCA1, and IGF1 (*n* = 8 different normal human donors). Gene induction was measured by RT-qPCR (Methods) by the ΔΔCt method with HPRT as housekeeping control. Equivalence of HPRT, GAPDH, and ACTAB as housekeepers was established. Mean ± SE, *each induction was statistically significant (ANOVA, *P* < 0.05, with Holm–Sidak correction, after normality testing by Shapiro–Wilk). *X*-axes, time in days (log scale). (*B*) hMDM were transfected with si-RNA to knock down ATF1. *X*-axis, si-RNA transfection of hMDM; *Y*-axis, ATF1 mRNA levels by RT-qPCR (Methods) by the ΔΔCt method with HPRT as housekeeping control, mean ± SE, *P < 0.05, Student’s *t*-test, *n* = 5 different human donors. (*C*) Metformin induces mRNA for HMOX1 in hMDM in control-transfected macrophages, but not in ATF1-siRNA-transfected macrophages (mean ± SE *Student’s *t*-test *P* < 0.05, *n* = 5 different human donors). (*D*) Metformin induces binding of p-ATF1 to the HMOX1 enhancer at the CRE site at −4200 bp in hMDM. Chromatin immunoprecipitation was performed as before, HMOX1 enhancer enrichment quantified by qPCR as before and expressed as fold enrichment and normalised to the IgG control (mean ± SE, *Student’s *t*-test *P* < 0.05, *n* = 5 different human donors). (*E*) FASL expression in hMDM at culture Day 8, measured by RT-qPCR. *Y*-axis, FASL fold induction measured by the ΔΔCt method (mean ± SE, *n* = 8 different human donors, *ANOVA *P* < 0.05, with Holm–Sidak correction and normality testing by Shapiro–Wilk). (*F*) CCR5 expression is induced in hMDM by loading with OxLDL, and reversed by metformin treatment. *Y*-axis, CCR5 fold induction by qRT-PCR and ΔΔCt method (*P < 0.05, *t*-test, *n* = 4 different human donors). (*G*) Metformin-enhanced phagocytosis in hMDM. *Y*-axis, phagocytic index for each phagocytosable particle, defined as number of particles left after washing divided by number of phagocytes (counted by nuclear DAPI stain). Expressed as mean ± SE *n* = 9, *passes significance, *P* < 0.002 paired Students’ *t*-test (beads), *P* < 0.002 Wilcoxon (ApC), *P* < 0.03 Wilcoxon (OxLDL). (*H*) Principal component analysis of microarray analysis of metformin versus vehicle control treatment of cultured hMDM (*n* = 4 different human donors) with arrows showing a consistent small effect of metformin in the same direction in each of the donors. Each donor is represented by a pair of points. (*I*) Hierarchical clustering and heat-map of microarray data of hMDM metformin treatment. The donors tended to cluster together. The genes that were significantly modulated by metformin were assessed in greater depth. (*J*) Venn diagram comparing genes modulated by heme, metformin, or both (intersection) on microarray analysis of over-represented TFBS in each subset of genes indicated likely differences in signalling and transcription factor usage. (*K*) Classification of top metformin-modulated genes by Gene Ontology from microarray analysis. Many of the genes encoded enzymes in intermediary metabolism, cell signalling, or leucocyte activation. The largest single category was unknown function. (*L*) Metformin induces NAMPT. NAMPT was one of the few unanticipated metformin-modulated protein-coding genes and was validated by qPCR. *Y*-axis: fold induction by RT-qPCR and −2ΔΔCt method (mean ± SE *P<0.05, ANOVA, Holm–Sidak adjustment *n* = 8 different human donors, normality tested by Shapiro–Wilk).

Next, hMDM were assessed (*Figure 4* and not shown). Metformin (10 μM) induced *ATF1*, *HMOX1*, *SOCS1*, *APOE*, *ABCA1*, *APOE*, *IGF1, ATF1*, *SOCS1*, *LXRA*, *LXRB IGF1*, and *PDGF* (*Figure 4A* and not shown). Therefore, the mBMM accurately reflected hMDM, indicating species conservation. Metformin-induced *HMOX1* was inhibited by si-*ATF1* gene knock-down in hMDM (*Figure*4*B and C*). A chromatin immunoprecipitation experiment showed that metformin initiated ATF1 binding to the *HMOX1* enhancer at approximately −4200 bp from the transcriptional start (*Figure 4D*). This is the same site that we previously defined as heme-responsive.^18^ That implies that the metformin-regulated activation is very similar to the heme-regulated activation.

### 3.4 Metformin inhibits OxLDL-induced expression of pathogenic genes by hMDM

Metformin suppresses hMDM and mBMM foam cell formation via AMPK.^17^ We assessed the effect of this on gene expression. Genes whose expression was increased in macrophages from human ruptured plaques were identified from the literature.^41^ Their modulation by OxLDL was validated by qPCR (not shown). The effect of combinations of OxLDL and metformin on gene expression in hMDM was measured (*Figure*4*E and F*). OxLDL-induced *FASL* and *CCR5*, and this induction was antagonised by metformin (*Figure*4*E and F*). Metformin also suppressed macrophage phagocytosis of OxLDL, polystyrene beads and apoptotic cells, which are scavenger receptor ligands, but not IgG-coated beads, which are ligands for Fc-receptors (*Figure 4G*).

We next asked whether metformin and heme had similar effects across the whole transcriptome. This was done by comparing metformin-modulated gene expression with our published heme dataset, using the same microarray system (*Figure 4H*–*K*). This showed significant similarity between heme-induced genes and metformin-induced genes (*Figure 4J*). Genes that were induced by both heme and metformin had enrichment of transcription factor binding sites (TFBS) for ATF1 [cyclic-AMP response elements (CRE)]. Genes that were induced by metformin but not by heme had enrichment for TFBS for the Forkhead transcription factor family. Heme-specific genes had enrichment of TFBS for NFE2L2 (Nrf2), the oxidative-stress responsive antioxidant transcription factor. GO analysis showed selective enrichment for genes for leucocyte activation, cell signalling and intermediary metabolism (*Figure 4J*). Many of these genes had been validated by qPCR before performing microarray. Some metformin-induced genes were long non-coding RNA species of unknown function. A novel target enzyme, *NAMPT*, was validated by qPCR (*Figure 4L*), Therefore, transcriptomics and qPCR showed that metformin induces an M2-like macrophage phenotype, overlapping with Mhem, in an ATF1-dependent manner.

### 3.5 Metformin induces M2-like genes in hMDM via ATF1

**Figure 5:**
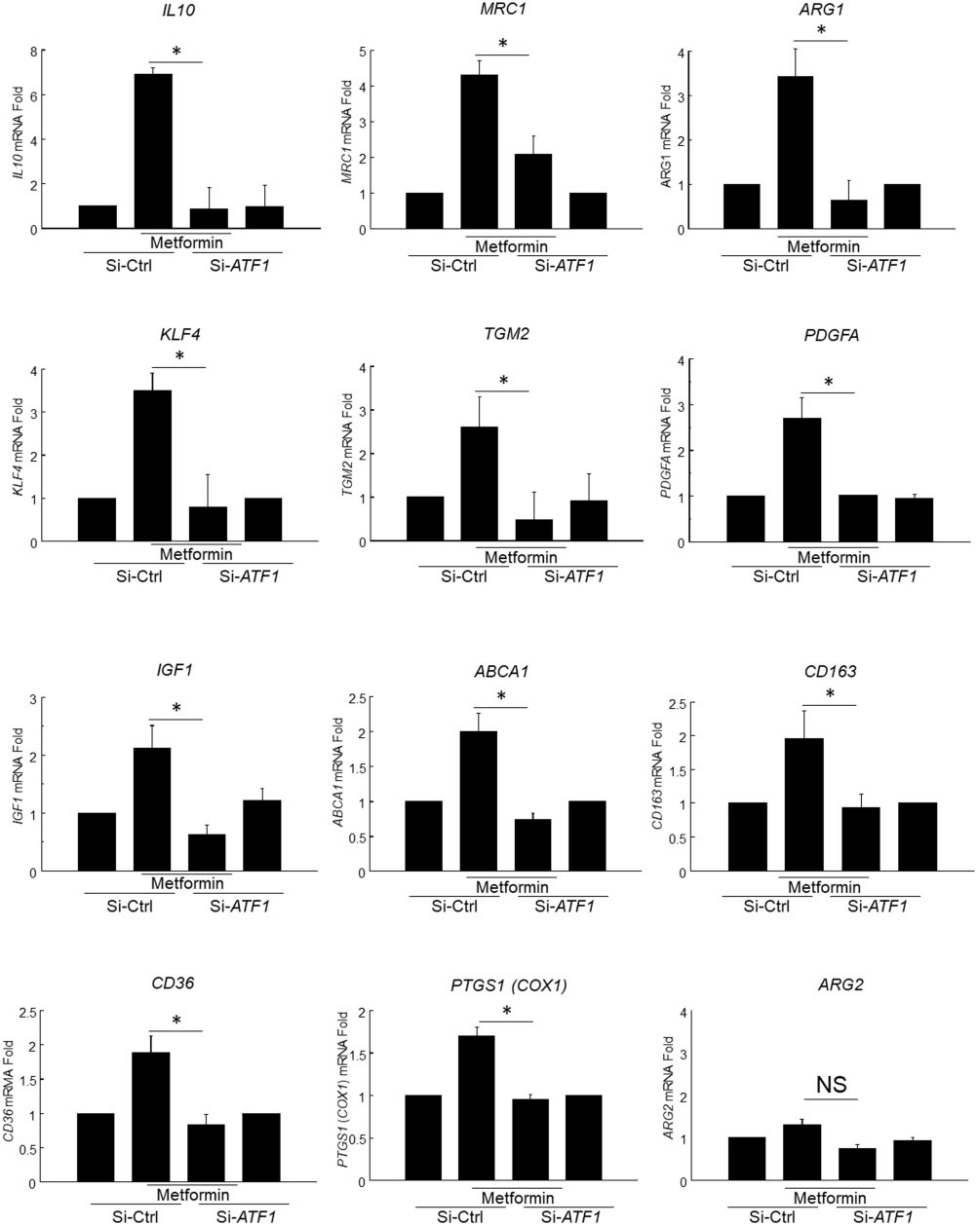
Metformin induces M2-like atheroprotective genes in hMDM. Panel of RT-qPCR experiments in hMDM treated with combinations of ATF1-knockdown and metformin. Genes are arranged by fold induction. Each gene name is in line with HGNC approved nomenclature, as indicated. Each *y*-axis, fold gene induction from −2ΔΔCt method (mean ± SE *P < 0.05, ANOVA, Holm–Sidak adjustment *n* = 8 different human donors, normality tested by Shapiro–Wilk. Si-ATF1, pretreatment with ATF1-siRNA as in methods; Si-Ctrl, control si-RNA. Metformin, metformin 10 μM 48 h.

Next, ATF1-dependence of metformin-responses in hMDM was assessed, using si-ATF1^18^ (*Figure 5*). Metformin induced the signature M2 genes *IL10*, *ARG1*, *MRC1*, *CD163*, *KLF4*, *TGM*. Metformin induced the growth factors *PDGF* and *IGF1*, and the lipid-clearance genes *CD36* and *ABCA1*. Induction of each of these was inhibited by si-ATF1, indicating that induction of these genes by metformin was ATF1-dependent. Induction of mouse M2 genes was also *Atf1*-dependent (Supplementary material online, *Figure SIV*). The gene modulation was accompanied by increases in the anti-atheroprotective eicosanoids PGI_2_ and RvD_1_ and reduction in the pro-atherogenic eicosanoid LTB_4_ (Supplementary material online, *Figures SV* and *SVI*).

### 3.6 Metformin activates macrophage AMPK/ATF1/HO-1 signalling and promotes an M2-like macrophage phenotype in the atherosclerotic plaque

**Figure 6:**
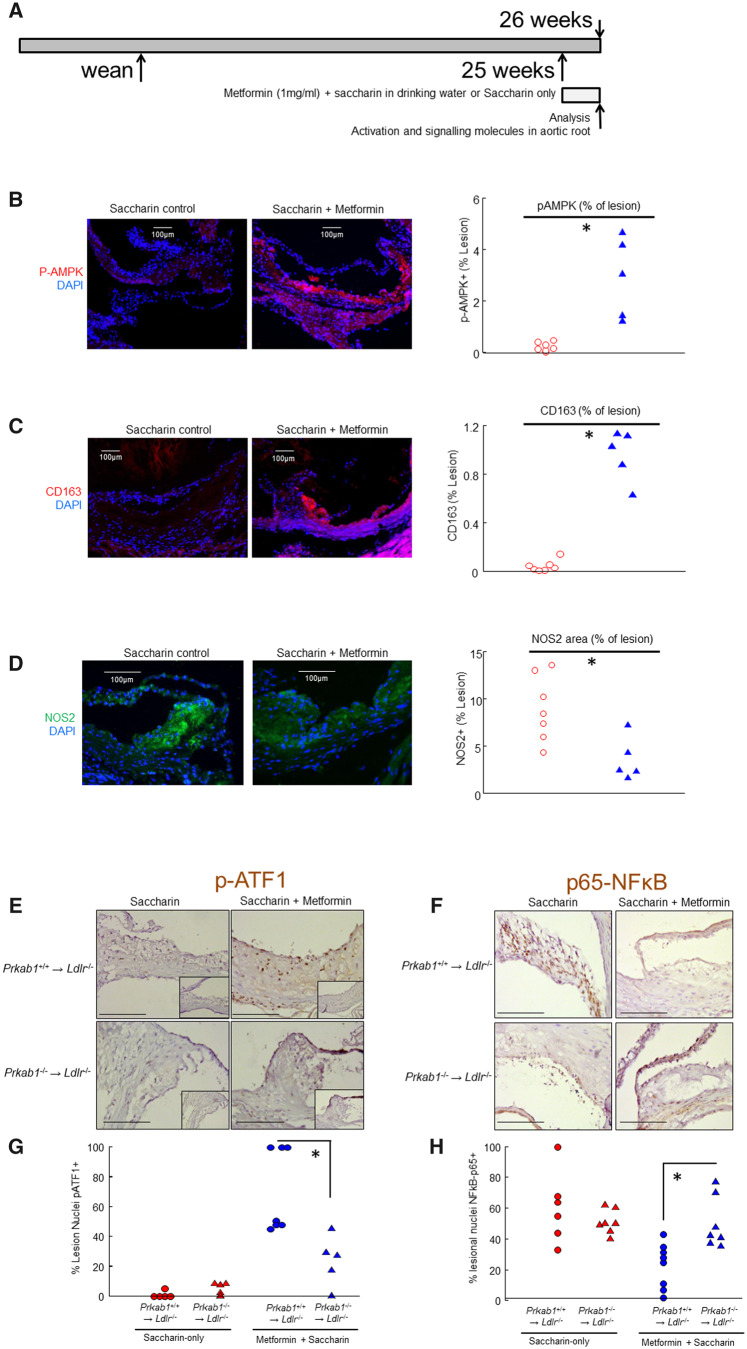
Metformin modulates signalling and decreases inflammation in the aortic root. (*A*) Graphical experimental protocol showing short duration of metformin treatment and increased age of commencement. (*B*) Representative micrographs for p-AMPK activation and quantification by planimetry. Scalebar = 100 μm. *P < 0.05, Wilcoxon rank sum test. Each point represents a different mouse, *n* = 6 mice (saccharin) *n* = 5 mice (metformin). (*C*) Representative micrographs for CD163 expression and quantification by planimetry. Scalebar = 100 μm. *P < 0.05, Wilcoxon rank sum test. Each point represents a different mouse, *n* = 7 mice (saccharin) *n* = 5 mice (metformin). (*D*) Representative micrographs for iNOS (NOS2) expression and quantification by planimetry. Scalebar = 100 μm. *P < 0.05, Wilcoxon rank sum test. Each point represents a different mouse, *n* = 7 mice (saccharin) *n* = 5 mice (metformin). (*E*) Immunoperoxidase for p-ATF1 in sections of the BMT cohort. Representative micrographs. Scalebar = 100 μm. Transplant status and oral treatment are as indicated. (*F*) Immunoperoxidase for p65-NFκB, in sections of aortic root of the cohort BMT from AMPK-deficient or littermate donors. Representative micrographs. Scalebar = 100 μm. Transplant status and oral treatment are as indicated. (*G*) Quantification of p-ATF1 staining of lesions as shown in *E*, calculated as the number of nuclei immunopositive for p-ATF1 relative to total lesional nuclei. Transplant marrow genotype and treatment are as shown. *Wilcoxon–Mann–Whitney *P* < 0.003 for indicated comparison, *n* = 5–7 mice, individual mice are represented by different points as shown. (*H*) Quantification of p65-NFκB staining of lesions as shown in E, calculated as the number of nuclei immunopositive for p65-NFκB relative to total lesional nuclei. Transplant marrow genotype and treatment are as shown. *Student’s *t*-test *P* < 0.005 for indicated comparison, *n* = 5–7 mice, individual mice are represented by different points as shown.

Because metformin had almost completely abrogated lesional macrophage accumulation, a different strategy was adopted to examine lesional macrophage signalling and gene modulation *in vivo. Ldlr*^−/−^ mice with established lesions on chow were treated with short-term oral metformin, at age 24 weeks old for 1 week (*Figure 6A*). In this protocol, metformin-induced lesional expression of pAMPK, pATF1, HO-1, CD163, and LXR (*Figure 6B, C, and E*, Supplementary material online, *Figures SVII* and *SVIII* and not shown). Metformin suppressed NOS2 (iNOS) and NF-κBp65 expression and nuclear translocation (*Figure 6D*, *F* and not shown). This indicated that metformin co-ordinately activated macrophage resolution properties and countered atherogenic activation.

To assess macrophage colocalization and AMPK-dependence of metformin-induced genes, lesions from the BMT cohort were also immunostained for HO-1 and CD163 and imaged by confocal microscopy. This demonstrated that metformin-induced lesional macrophage p-ATF1, HO-1 and CD163, and suppressed NOS2 and NF-κBp65 expression and nuclear translocation, dependent on AMPK (*Figure*6*E and F*, Supplementary material online, *Figures SVII* and *SVIII* and not shown).

### 3.7 Metformin does not modulate circulating monocyte chemokine receptors

We next asked whether metformin was likely to have been effective by modulating peripheral blood monocytes. Since hyperlipidaemia modulates monocyte subsets,^42^ and CCR2, CCR5, and CX3CR1 chemokine receptors mediate plaque monocyte recruitment,^43^ these were assessed by flow cytometry (Supplementary material online, *Figure SIX*). Metformin-treatment did not alter the major monocyte subsets, nor their expression of CCR2, CCR5, and CX3CR1. These data excluded a major role for monocyte-modulation in atherosclerosis prevention by metformin in this model.

### 3.8 Long-term treatment of established lesions by metformin alters atherosclerotic lesion composition

Since metformin treatment often starts in patients with known disease, its effect on established plaques was examined. Mice were commenced on metformin, using the same oral dosing and analysis as before, but beginning at 25 weeks and lasting for 15 weeks (Supplementary material online, *Figure SXA*). Under these conditions, metformin did not reduce overall lesion size (Supplementary material online, *Figure SXB*), but consistently stimulated the formation of a layer of VSMCs between the endothelium and the plaque macrophages (Supplementary material online, *Figure SXB* and *C*). These approximated an early fibrous cap, insofar as development in a mouse lesion would allow (Supplementary material online, *Figure SXC*).

Given that metformin-induced macrophage *Igf1* (Supplementary material online, *Figure SXD*, *Figures*3*A and B*, and 4), lesions were next immunostained for p-IGFR1. This is the auto-phosphorylated form of the IGF1 receptor that mediates cell signalling. The immunohistochemistry was validated on formaldehyde-fixed cultured cells (Supplementary material online, *Figure SIXE*), and then applied to tissue sections from the *in vivo* experiment. This showed that metformin-induced plaque VSMCs were immunopositive for p-IGF1R (Supplementary material online, *Figure SIXF*). This indicated that IGF1 was bioactive in the lesions of metformin-treated mice, possibly promoting VSMC survival and growth. Indeed, metformin suppressed macrophage-induced VSMC apoptosis *in vitro*, reversed by neutralizing anti-IGF antibodies (Supplementary material online, *Figure SIXG*). Therefore, metformin may have a stabilizing effect in established atherosclerotic lesions.

## 4. Discussion

Inhibition of diabetic macrovascular disease by metformin had already been suspected.^8^^,^^44–47^ These data demonstrate direct suppression of euglycaemic atherosclerosis. The data also add an *in vivo* mechanism mediated by haematopoietic AMPK. Moreover, *in vivo* and *in vitro* AMP-dependent metformin-mediated gene regulation were correlated and support M2-like gene modulation. The *in vitro* data show that the gene regulation was mediated by the transcription factor ATF1, which is a novel mechanism. Because metformin is already a first-line oral antidiabetic, these findings are clinically relevant.

Metformin and atorvastatin co-treatment synergistically reduced atherosclerosis in high-fat fed rabbits.^44^ In fat-fed *Apoe*^−/−^ mice infused with Angiotensin-II, metformin was hypoglycaemic as well as suppressing atherosclerosis.^46^ In *Apoe*^−/−^ mice with infusions of Ang-II that induced aortic aneurysms, metformin suppressed aneurysm formation.^45^ In *Apoe*^−/−^ mice fed a high-fat diet, metformin and other AMPK-activators suppressed peripheral monocyte count.^47^ Thus in each of these studies, other effects of metformin acted as a confounder for assessment of atherogenesis. Our focus on a mild model and on AMPK allowed us to define a clinically relevant specific mechanism. Moreover, AMPK was not required for the hypoglycaemic effects of metformin *in vivo.*^13^ That is, the signature drug effect of metformin appears to be AMPK-independent. In contrast, we have positively identified the importance of AMPK (*Prkab1*) in macrophages *in vitro* and in haematopoietic cells in atherogenesis *in vivo*. This adds to the understanding of the pharmacology of metformin.

If these human *in vitro* and murine *in vivo* effects have relevance, one might expect some signal in clinical data.^27^ This is indeed the case, as seen in UKPDS34.^7^^,^^27^ The difficulty of placebo-controlled studies in human diabetic patients complicates interpretation of trials.^5–9^^,^^27^ Since it is not ethical to leave diabetics untreated, the randomized trials must compare metformin with other hypoglycaemic agents. It is therefore difficult to know conclusively whether the apparent benefit of metformin relates to metformin decreasing atherosclerosis or off-target actions of other drugs.^5–9^ For example, one of the largest and highest quality trials is UKPDS34.^7^^,^^27^ This is a double-blind prospective randomized controlled trial. In it, patients are randomized to aggressive glucose-lowering with either an insulin/sulphonylurea-based regime or a metformin-based regime. Importantly, each regime established equivalent hypoglycaemia.^7^ Patients were followed up and MACE were counted.^7^ This is the gold-standard endpoint, comprising myocardial infarction, unstable angina, peripheral arterial occlusion and stroke.^7^ Metformin significantly reduced MACE, with a progressive significance over a 10–15 years span.^7^ In one more recent study, metformin and placebo were randomized in a non-diabetic study group of only six patients, and carotid intima: media thickness was measured after 2 years.^48^ There was no significant difference. This may be due to the relatively early lesions, lack of assessment of plaque vulnerability or clinical events, or to the early timepoint, or to low study numbers. We therefore suggest that we have added significantly to the literature, by providing a novel mechanism for a drug in common clinical usage.

A decade ago, we described a macrophage phenotype that is associated with intraplaque haemorrhage in fatal ruptured human coronary lesions.^20^ Studying these in greater depth indicated that they had putatively atheroprotective properties.^20^ Our data then revealed interlinking of iron-regulatory and lipid regulatory gene networks by ATF1.^18^ Our data then showed the ATF1 was activated by AMPK.^17^^,^^18^ Since metformin may also activate AMPK, this study was an obvious next step.

The mildness of the chow-fed *Ldlr*^−/−^ model was an asset that probably revealed the effect of metformin. Previous studies included significant confounders such as Ang-II treatment, co-treatment with statins, glucose reduction and monocytosis.^44–47^ We selected chow-fed *Ldlr*^−/−^ mice because their relative metabolic normality allowed a better baseline to study directly atheroprotective effects without confounders. In particular, glucose levels were within normal limits for strain, and there was only mild steatohepatitis (not shown). Since the lesions were simple, comprising small collections of macrophages, the model facilitated clear experimental data. Moreover, whilst lesions at this stage do not have intraplaque haemorrhage, we are taking lessons from our previous studies on responses to intraplaque haemorrhage^49^ in order to repurpose an exogenous protective drug.

The BMT experiment was important in two respects. First, it showed that metformin directly and profoundly suppresses the development of atherosclerosis, by a direct effect on haematopoietic cells. Secondly, it demonstrated a requirement for haematopoietic AMPK for the metformin effects *in vivo*. These data may explain the apparent unique effect of metformin as an antidiabetic drug that prevents MACE.

The human and mouse time-course qPCR data for metformin-induced gene expression were closely matched, indicating that mice are a representative model, adding to the validity of the *in vivo* experiments. This point was important, since the representativeness of murine inflammatory models is questioned.^50^^,^^51^ When human metformin responses were assessed transcriptomically in comparison with heme, there was significant overlap. This indicated that metformin does indeed partially reproduce the protective response to intraplaque haemorrhage. This common geneset had significant enrichment for CRE (ATF1 sites), also validating our views of the signalling. There were also many non-overlapping genes, indicating that responses to heme and metformin are distinct. Metformin-specific genes had increased numbers of Forkhead TFBS, which is consistent with the literature.^52^ In passing, the microarrays detected OCT1 (SLC22A1), a metformin transporter, explaining its effectiveness in macrophages.^18^ One of the most highly induced genes, NAMPT (visfatin) has been implicated in mitochondrial metabolism, cell survival, atherosclerosis susceptibility and lipid export,^53^ as well as renal preservation *in vivo*^54^ and will be a future point of study.

There are parallels in the molecular signalling and gene expression caused by heme and metformin. This is not an argument that the pharmacological effect of metformin is due to the pathophysiological event of intraplaque haemorrhage. Rather, the argument is that some protective effects of intraplaque haemorrhage may be reproduced by metformin. Engaging these mechanisms in advance of actual intraplaque haemorrhage may therefore be preventive.

The number of AMPK/ATF1 coordinated effector mechanisms prevented a full mechanistic dissection of each. However, control by AMPK, ATF1 and usually both, was shown. The number of mechanisms involved may contribute to the relative strength of overall *in vivo* effect. Others have shown the importance of each in atherosclerosis: IL-10,^55^ HO-1,^56^ CD163,^57^ ABCA1,^58^ LTB_4_ and RvD_1_,^59^ PGI_2_,^60^ and M2-like macrophages.^61^

## 5. Conclusions

Metformin directly protects against atherosclerosis, independently of normalization of lipids or glucose. It does so via haematopoietic AMPK *in vivo*. This contrasts with the hypoglycaemic effect, which is AMPK-independent.^13^*In vitro* metformin effects were also mediated by ATF1 and a macrophage M2-like gene expression. Targets included the antioxidant gene *HMOX-1*; lipid homeostasis genes such as *ABCA1*; M2-like genes such as *IL10* and *CD163*; growth factors and anti-atherosclerotic eicosanoids. Our data therefore support further experimental medicine studies of metformin in non-diabetic patients at cardiovascular risk.

## Data availability

The microarray datasets were uploaded to GEO (https://www.ncbi.nlm.nih.gov/geo/) and will be available after their curation. Further data underlying this article are available in the article and in its online supplementary material. Any remaining data underlying this article will be shared on reasonable request to the corresponding author.

**Translational perspective**

The work shows that oral antidiabetic drug metformin may suppress atherosclerotic lesion development via haematopoietic AMPK at clinically relevant concentrations, rather than via a hypoglycaemic effect. Activating transcription factor 1 may mediate induction of key atheroprotective genes by metformin. This suggests a mechanism for some of the effects of metformin. It has strong implications for a possible role as an atheroprotective agent beyond the context of diabetes. The data support a clinical trial of metformin in non-diabetic patients at high risk of atherosclerosis.

## Supplementary material

Supplementary material is available at *Cardiovascular Research* online.

**Conflict of interest:** none declared.

## Funding

The work was supported by the British Heart Foundation (BHF SCRF03 FS/13/12/30037 to J.J.B., also PG/15/57/31580, PG/17/71/33242). Some of the imaging was carried out with instruments in FILM facility, Imperial College London. The Facility for Imaging by Light Microscopy (FILM) at Imperial College London is part supported by funding from the Wellcome Trust (104931/Z/14/Z) and Biotechnology and Biological Sciences Research Council (BBSRC) (BB/L015129/1). This work was supported by the NIHR Imperial Biomedical Research Centre (BRC). The views expressed are those of the author(s) and not necessarily those of the NIHR or the Department of Health and Social Care.

## Supplementary Material

cvaa171_Supplementary_Data

## Notes

Time for primary review: 26 days
