# Supplementary material for: Metformin directly suppresses atherosclerosis in normoglycaemic mice via haematopoietic adenosine monophosphate-activated protein kinase
Source: Cardiovasc Res. 2020 Jun 25;117(5):1295–308. doi: 10.1093/cvr/cvaa171 (PMC8064441; doi:10.1093/cvr/cvaa171)
Supplement: cvaa171_Supplementary_Data [file cvaa171_supplementary_data.zip › Suppl/23-5-20 EHJ Figures Online.pptx]

## Slide 1
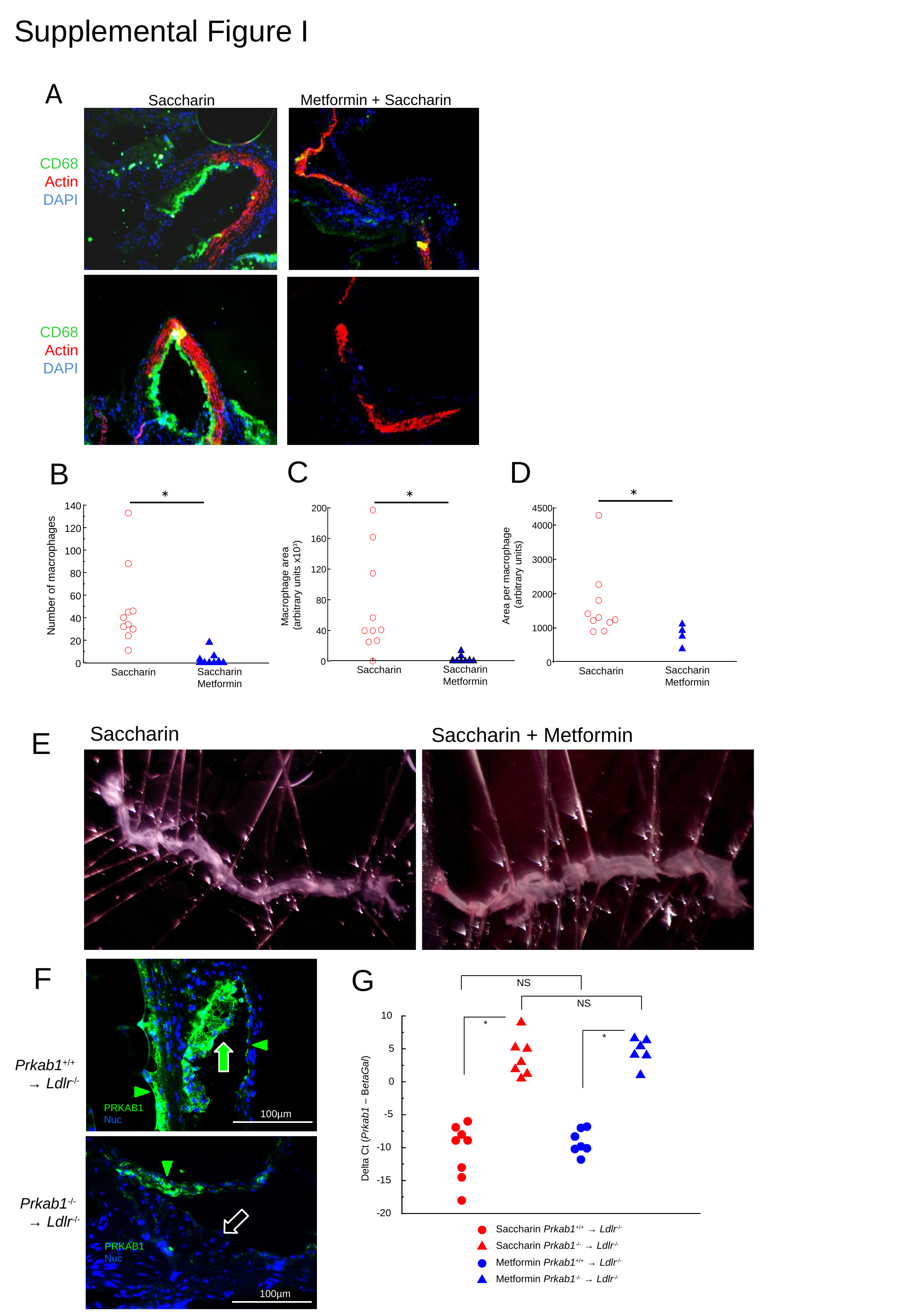

Supplemental Figure I
A
Metformin + Saccharin
Saccharin
CD68
Actin
DAPI
CD68
Actin
DAPI
C
D
B
*
*
*
140
120
100
80
Number of macrophages
60
40
20
0
Saccharin
Metformin
Saccharin
4500
200
160
120
Macrophage area
 (arbitrary units x103)
80
40
0
Saccharin
Metformin
Saccharin
4000
3000
Area per macrophage
(arbitrary units)
2000
1000
0
Saccharin
Metformin
Saccharin
Saccharin
Saccharin + Metformin
E
F
G
NS
NS
10
5
0
-5
Delta Ct (Prkab1 – BetaGal)
-10
-15
-20
*
*
Prkab1+/+
→ Ldlr-/-
PRKAB1 / Nuc
100µm
Prkab1-/-
→ Ldlr-/-
Saccharin Prkab1+/+ → Ldlr-/-
Saccharin Prkab1-/- → Ldlr-/-
Metformin Prkab1+/+ → Ldlr-/-
Metformin Prkab1-/- → Ldlr-/-
PRKAB1 / Nuc
100µm

## Slide 2
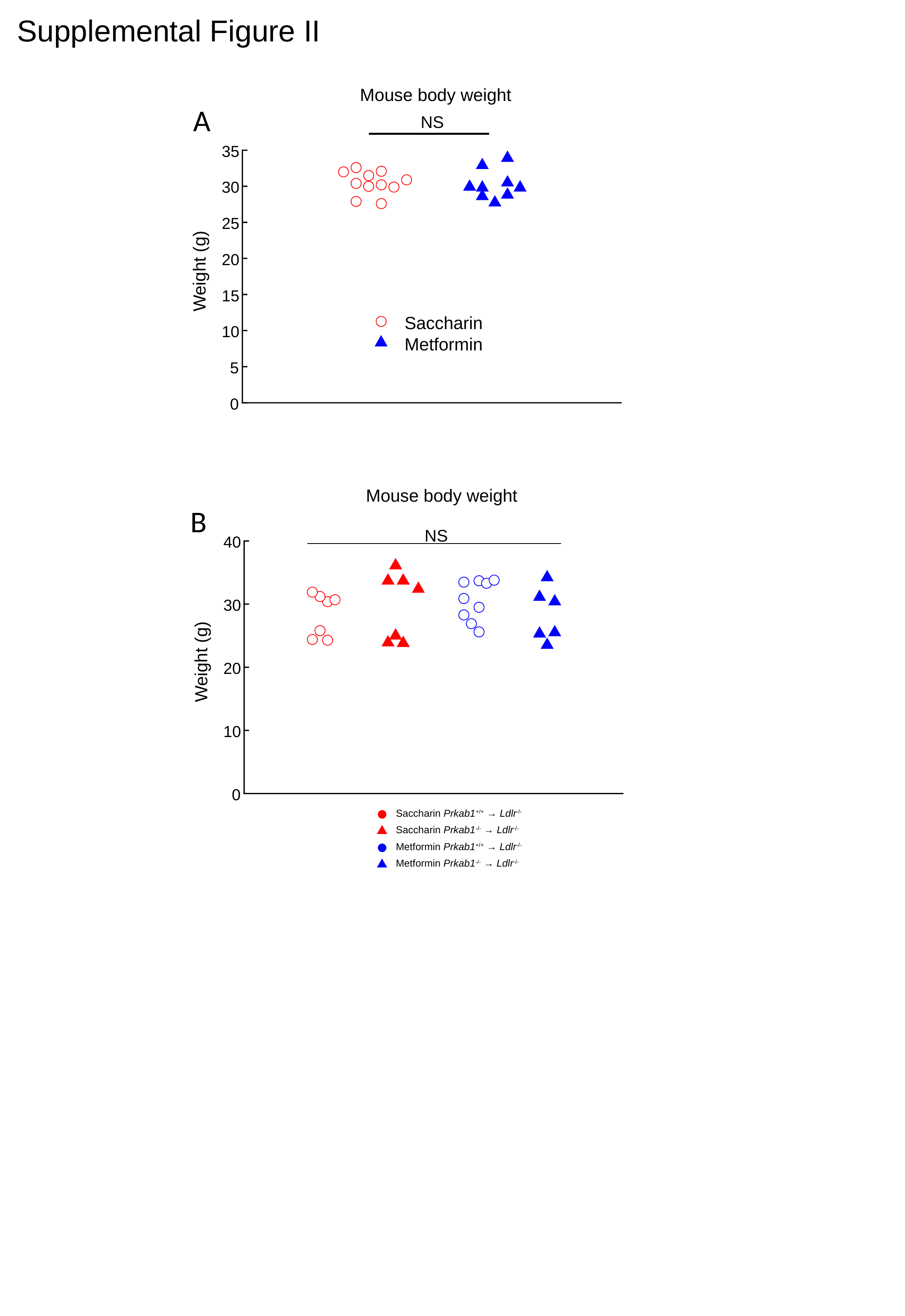

Supplemental Figure II
Mouse body weight
NS
35
30
25
20
Weight (g)
15
Saccharin
Metformin
10
5
0
A
Mouse body weight
NS
40
30
Weight (g)
20
10
0
Saccharin Prkab1+/+ → Ldlr-/-
Saccharin Prkab1-/- → Ldlr-/-
Metformin Prkab1+/+ → Ldlr-/-
Metformin Prkab1-/- → Ldlr-/-
B

## Slide 3
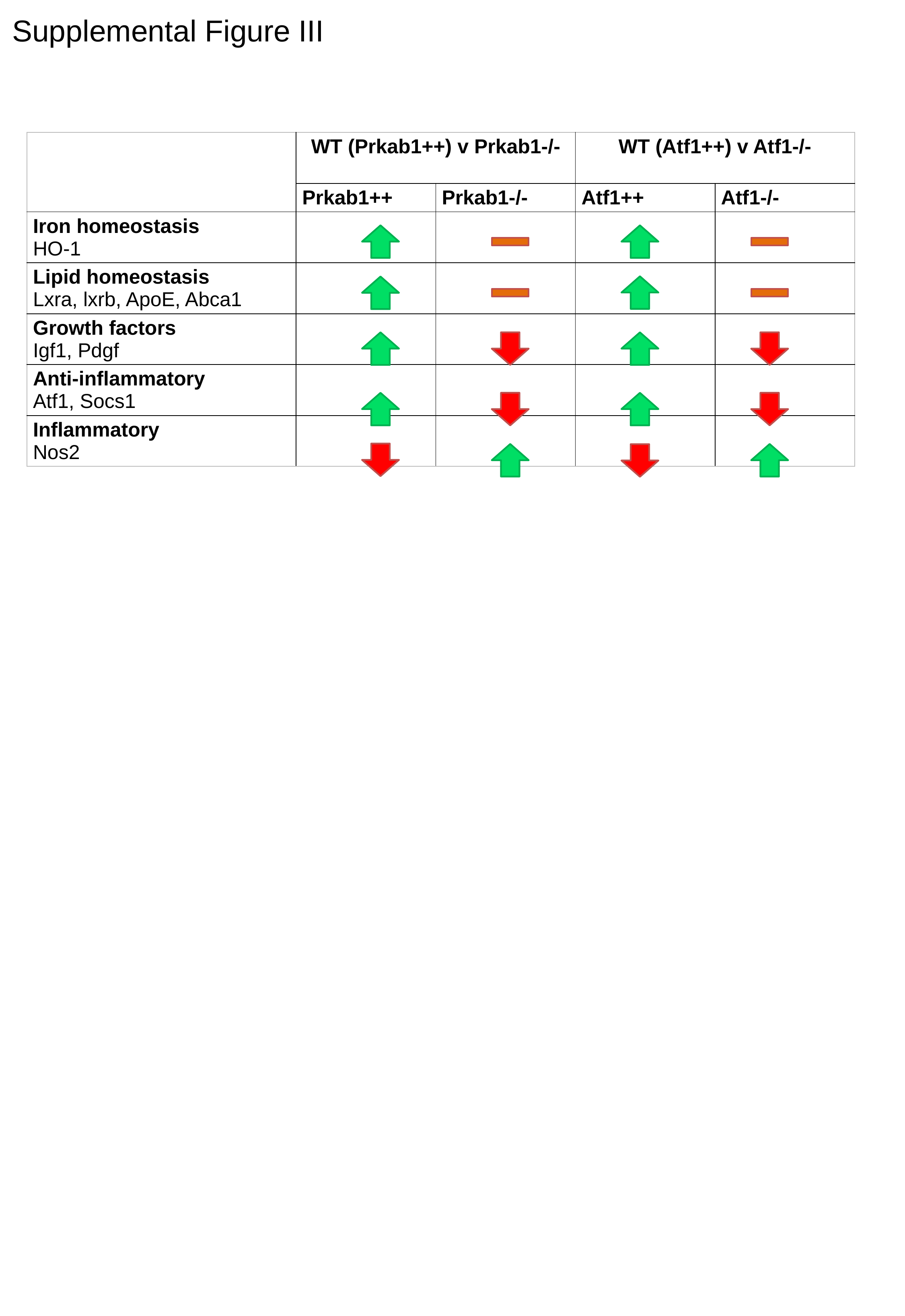

# Supplemental Figure III
| | WT (Prkab1++) v Prkab1-/- | | WT (Atf1++) v Atf1-/- | |
| --- | --- | --- | --- | --- |
| | Prkab1++ | Prkab1-/- | Atf1++ | Atf1-/- |
| Iron homeostasis HO-1 | | | | |
| Lipid homeostasis Lxra, lxrb, ApoE, Abca1 | | | | |
| Growth factors Igf1, Pdgf | | | | |
| Anti-inflammatory Atf1, Socs1 | | | | |
| Inflammatory Nos2 | | | | |

## Slide 4
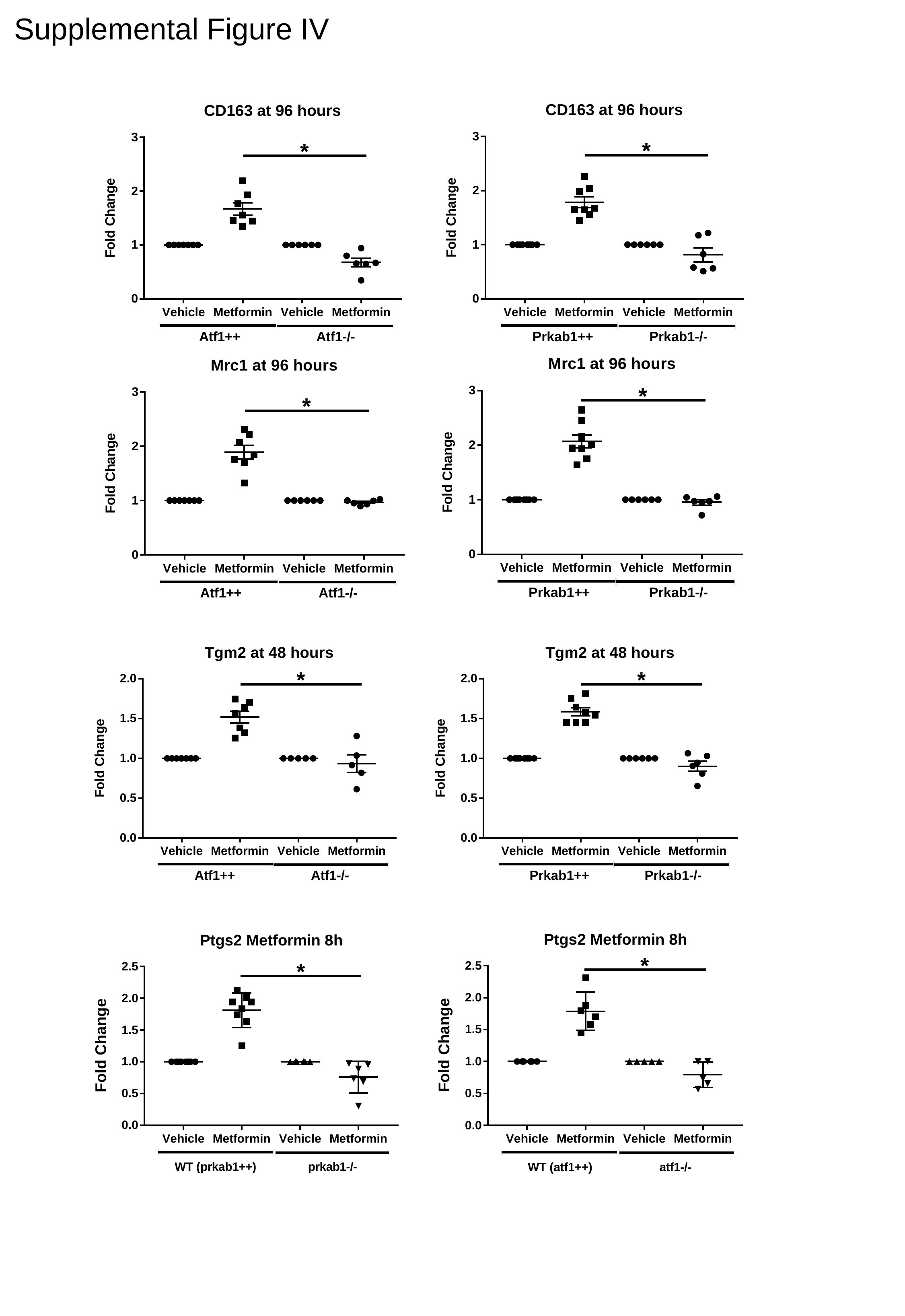

# Supplemental Figure IV

## Slide 5
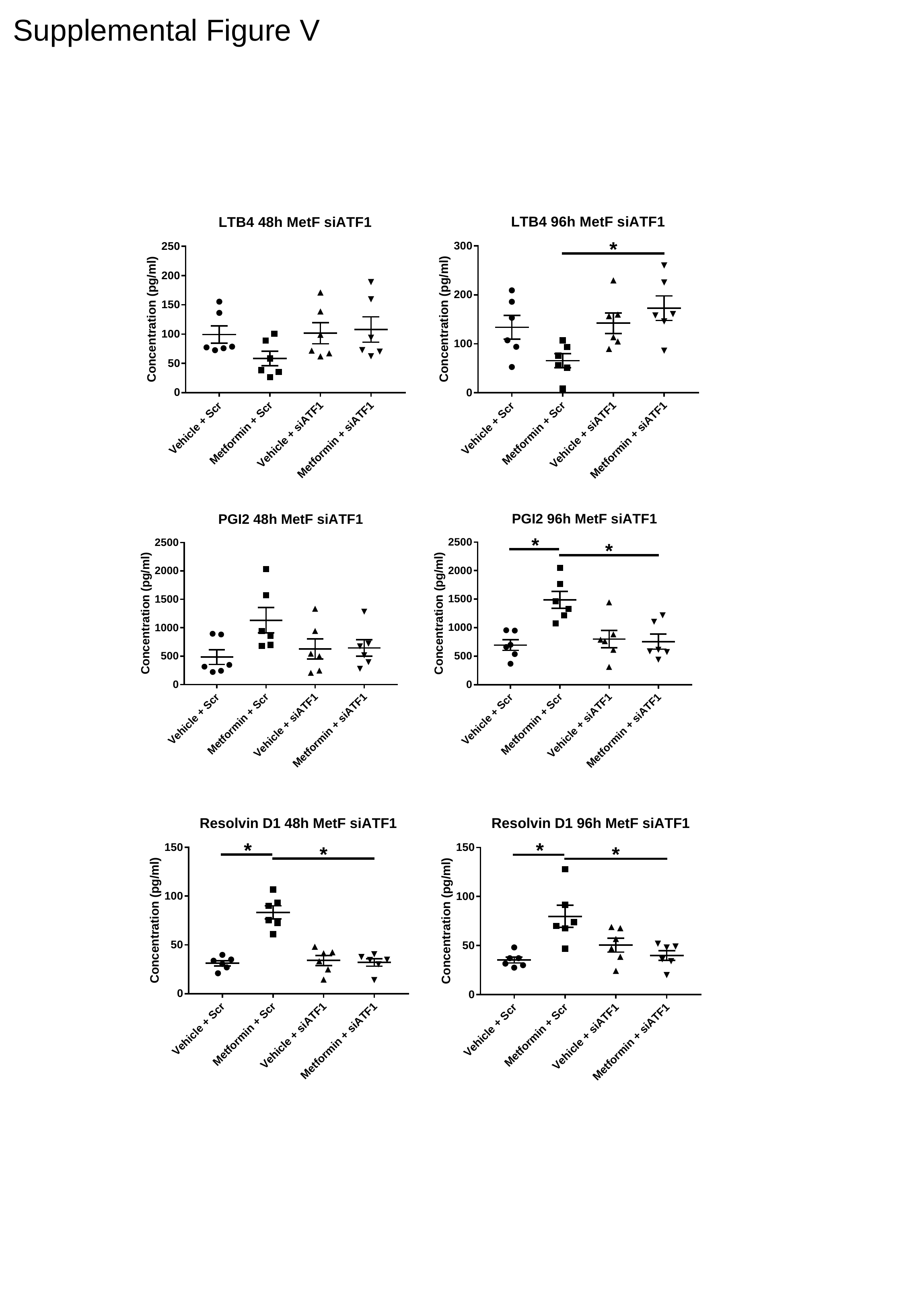

Supplemental Figure V

## Slide 6
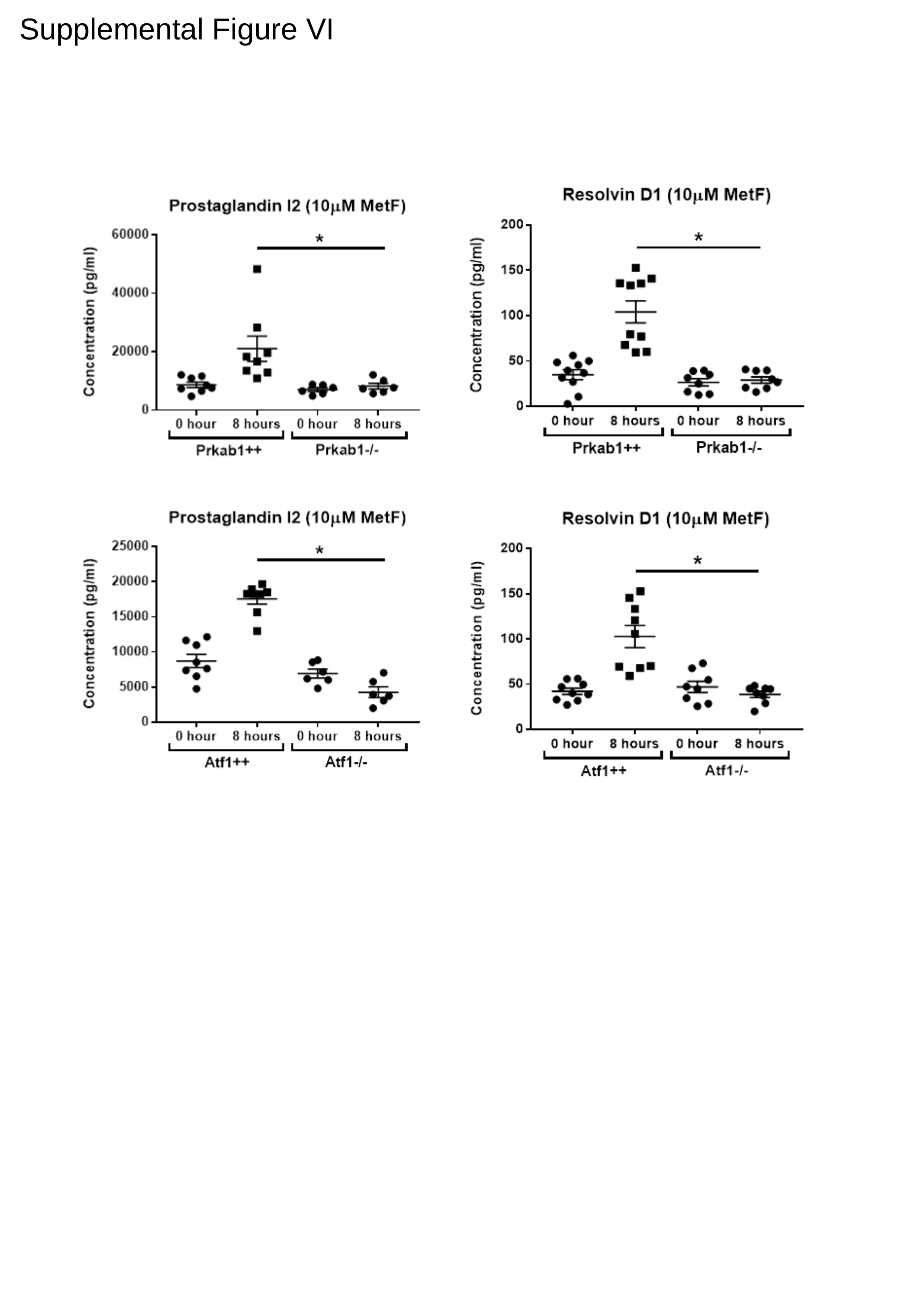

Supplemental Figure VI

## Slide 7
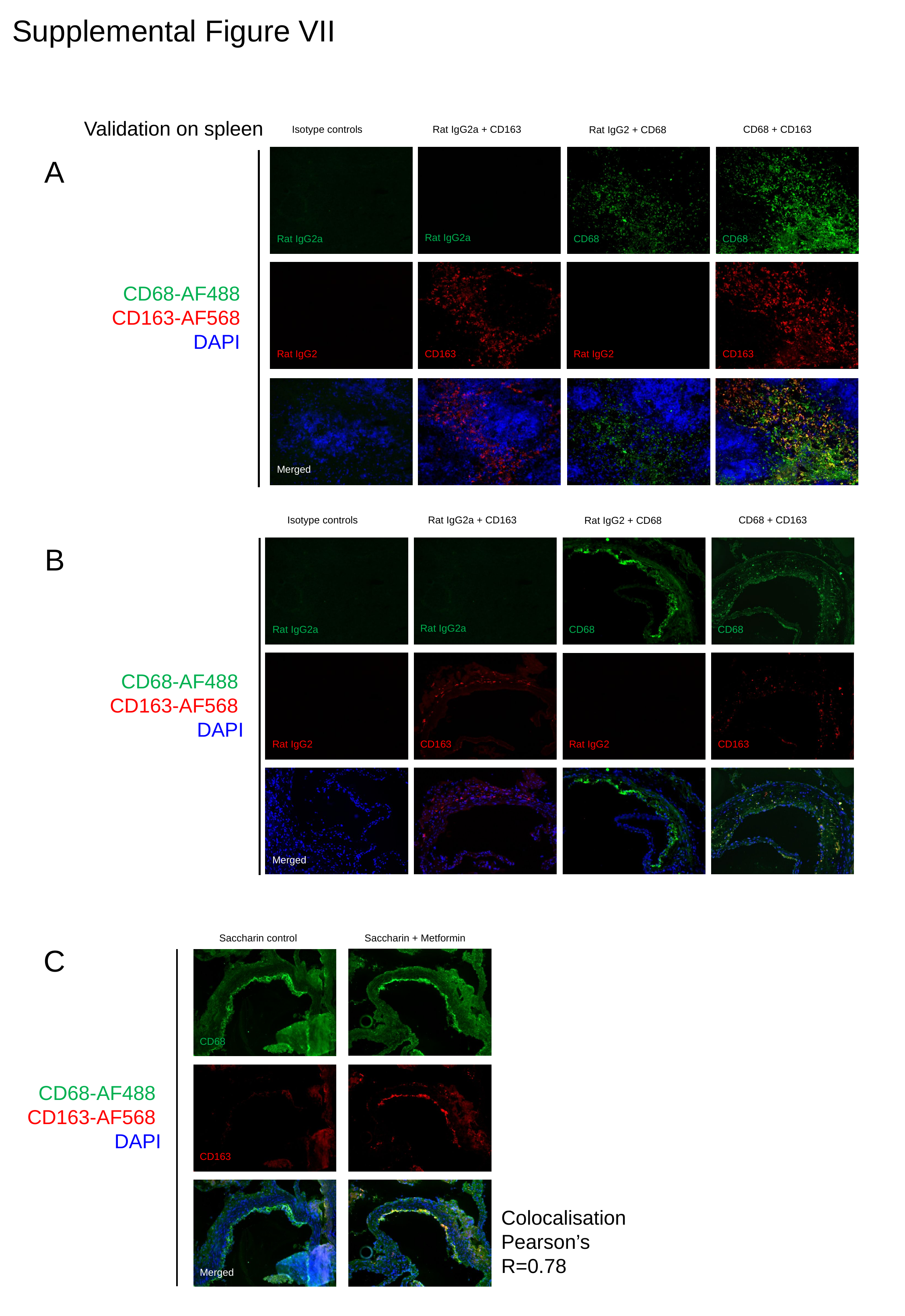

Supplemental Figure VII
Validation on spleen
Isotype controls
Rat IgG2a + CD163
CD68 + CD163
Rat IgG2 + CD68
Rat IgG2a
Rat IgG2a
CD68
CD68
Rat IgG2
CD163
Rat IgG2
CD163
Merged
CD68-AF488
CD163-AF568
DAPI
A
Isotype controls
Rat IgG2a + CD163
CD68 + CD163
Rat IgG2 + CD68
Rat IgG2a
Rat IgG2a
CD68
CD68
Rat IgG2
CD163
Rat IgG2
CD163
Merged
CD68-AF488
CD163-AF568
DAPI
B
Saccharin control
Saccharin + Metformin
CD68-AF488
CD163-AF568
DAPI
CD68
CD163
Merged
C
Colocalisation Pearson’s R=0.78

## Slide 8
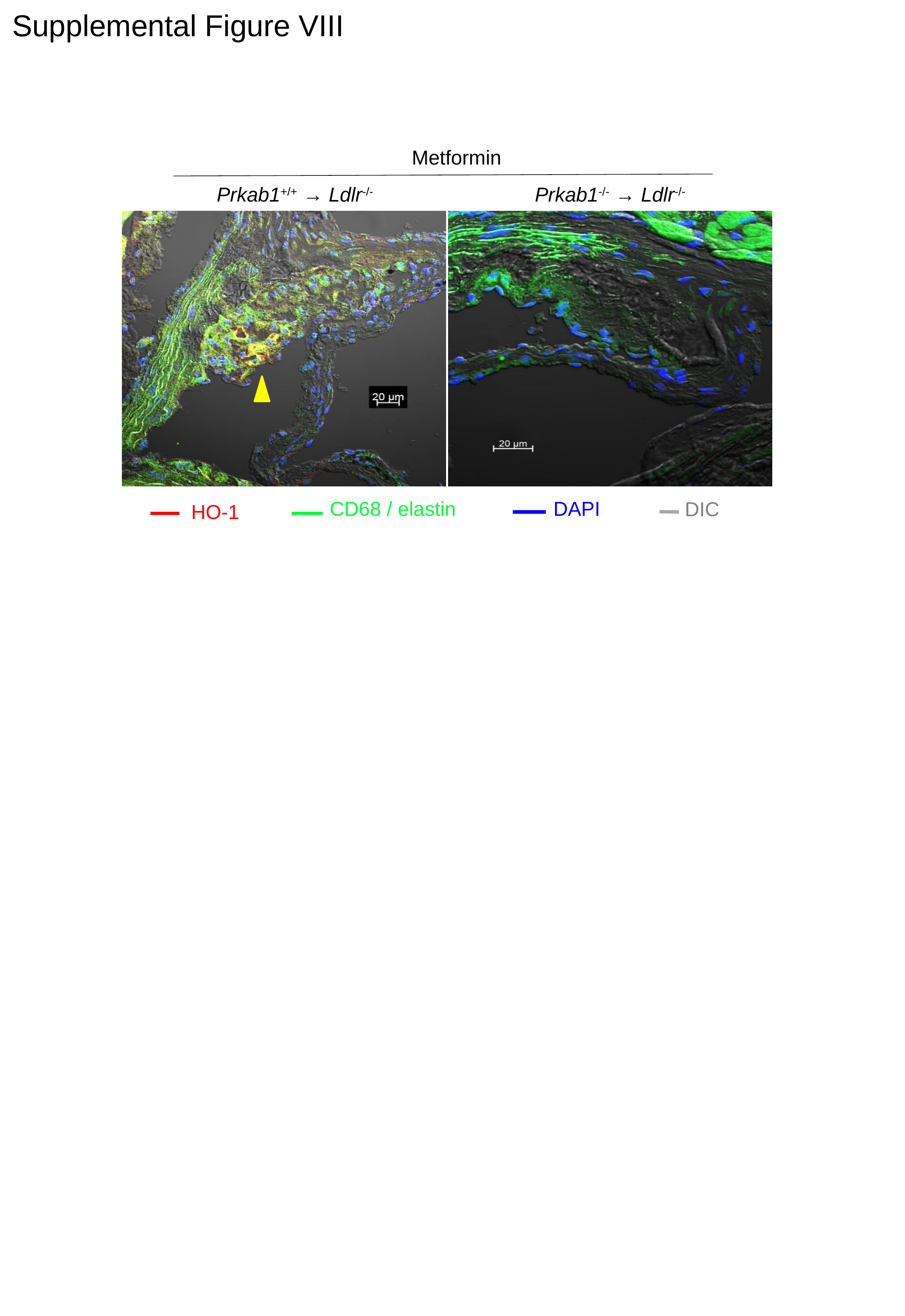

Supplemental Figure VIII
Metformin
Prkab1+/+ → Ldlr-/-
Prkab1-/- → Ldlr-/-
DAPI
CD68 / elastin
DIC
HO-1

## Slide 9
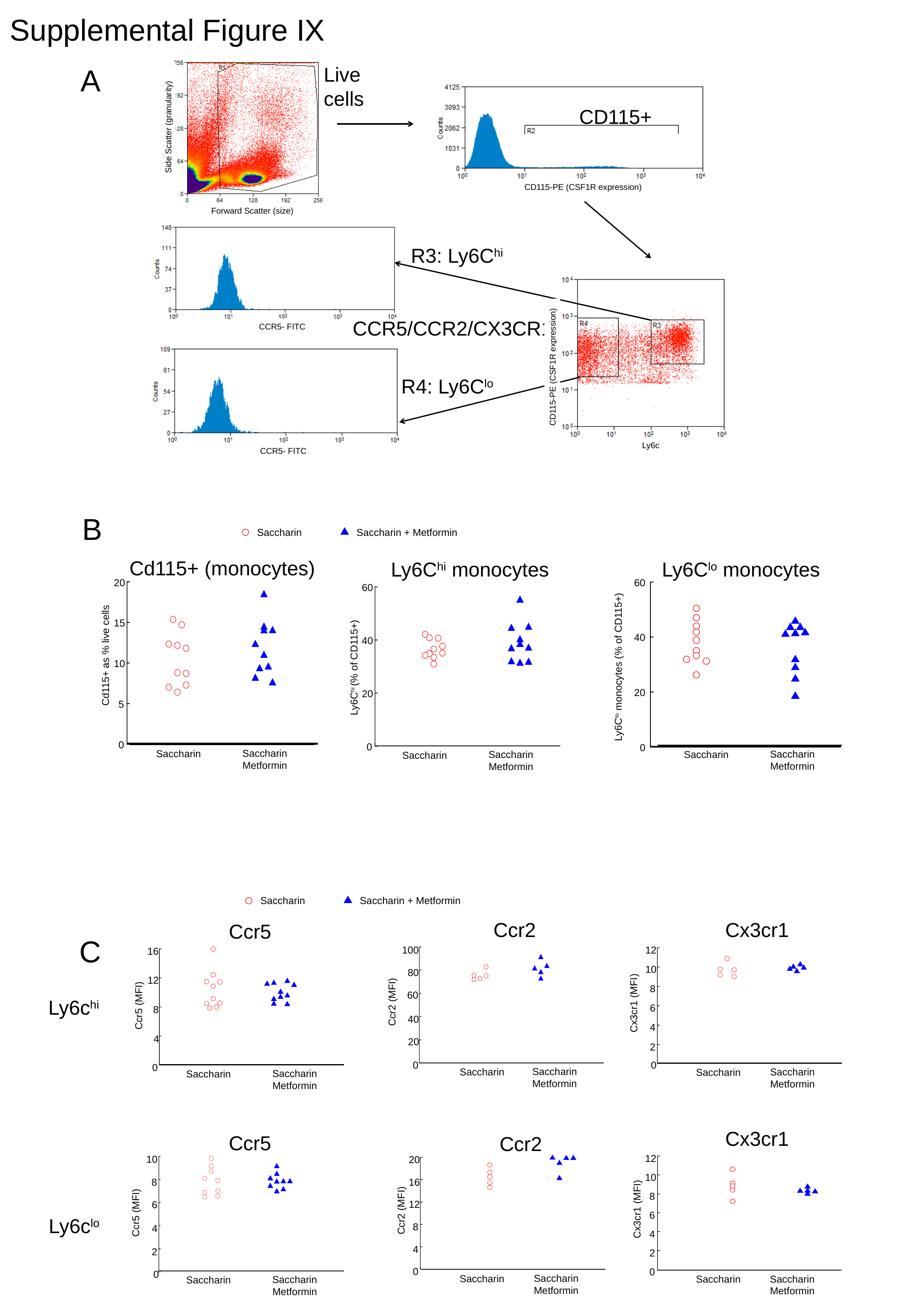

Supplemental Figure IX
Live
cells
CD115+
R3: Ly6Chi
CCR5/CCR2/CX3CR1
R4: Ly6Clo
A
Side Scatter (granularity)
CD115-PE (CSF1R expression)
Forward Scatter (size)
CCR5- FITC
CD115-PE (CSF1R expression)
Ly6c
CCR5- FITC
B
Saccharin
Saccharin + Metformin
Cd115+ (monocytes)
Ly6Chi monocytes
Ly6Clo monocytes
60
20
15
Cd115+ as % live cells
10
5
0
Saccharin
Metformin
Saccharin
60
40
40
Ly6Clo monocytes (% of CD115+)
Ly6Chi (% of CD115+)
20
20
0
0
Saccharin
Metformin
Saccharin
Saccharin
Metformin
Saccharin
Saccharin
Saccharin + Metformin
Cx3cr1
12
10
8
Cx3cr1 (MFI)
6
4
2
0
Saccharin
Metformin
Saccharin
Cx3cr1
12
10
8
Cx3cr1 (MFI)
6
4
2
0
Saccharin
Metformin
Saccharin
Ccr2
100
80
60
Ccr2 (MFI)
40
20
0
Saccharin
Metformin
Saccharin
Ccr2
20
16
12
Ccr2 (MFI)
8
4
0
Saccharin
Metformin
Saccharin
Ccr5
16
12
Ccr5 (MFI)
8
4
0
Saccharin
Metformin
Saccharin
Ccr5
10
8
6
Ccr5 (MFI)
4
2
0
Saccharin
Metformin
Saccharin
C
Ly6chi
Ly6clo

## Slide 10
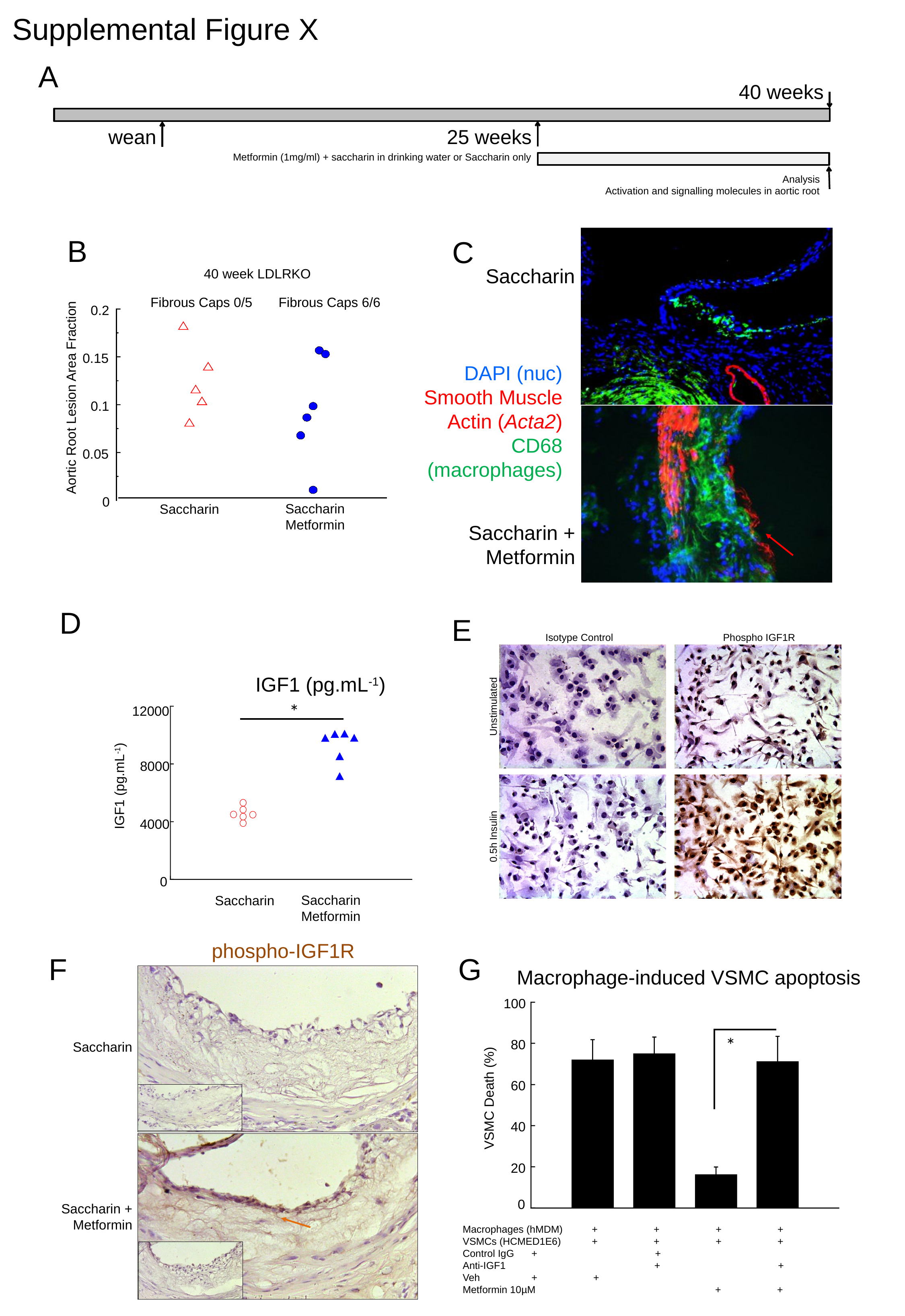

Supplemental Figure X
A
40 weeks
wean
25 weeks
Metformin (1mg/ml) + saccharin in drinking water or Saccharin only
Analysis
Activation and signalling molecules in aortic root
B
C
40 week LDLRKO
Fibrous Caps 0/5
Fibrous Caps 6/6
0.2
0.15
Aortic Root Lesion Area Fraction
0.1
0.05
0
Saccharin
Metformin
Saccharin
DAPI (nuc)
Smooth Muscle Actin (Acta2)
CD68 (macrophages)
Saccharin
Saccharin + Metformin
D
E
Isotype Control
Phospho IGF1R
Unstimulated
0.5h Insulin
IGF1 (pg.mL-1)
12000
8000
IGF1 (pg.mL-1)
4000
0
*
Saccharin
Metformin
Saccharin
phospho-IGF1R
F
Saccharin
Saccharin + Metformin
G
Macrophage-induced VSMC apoptosis
100
80
60
VSMC Death (%)
40
20
0
*
Macrophages (hMDM)	 + + + +
VSMCs (HCMED1E6) 	 + + + +
Control IgG	 + +
Anti-IGF1 + +
Veh	 + +
Metformin 10µM	 + +

## Slide 11
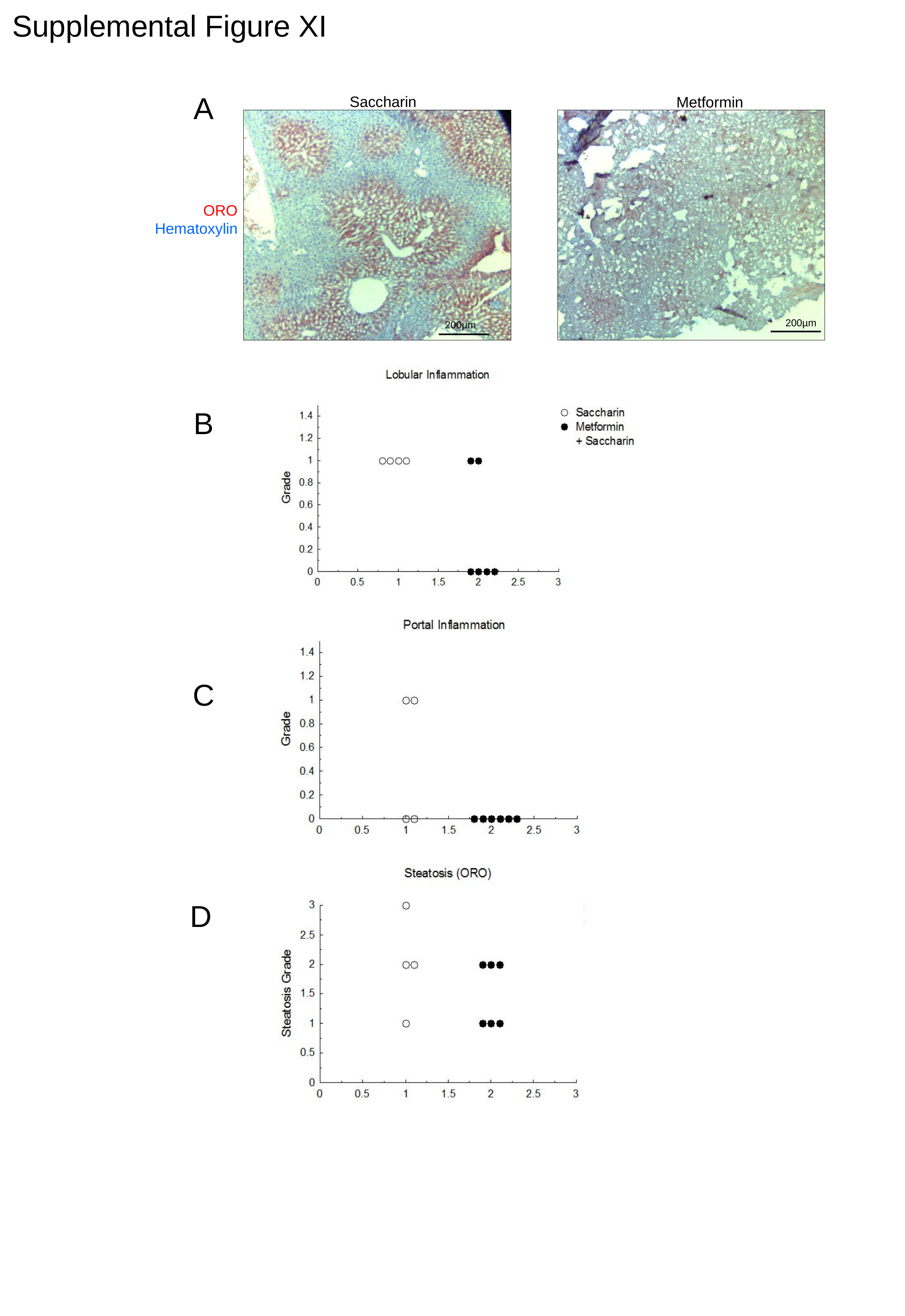

Supplemental Figure XI
A
Saccharin
Metformin
ORO
Hematoxylin
200µm
200µm
B
C
D
