# Supplementary material for: Metformin directly suppresses atherosclerosis in normoglycaemic mice via haematopoietic adenosine monophosphate-activated protein kinase
Source: Cardiovasc Res. 2020 Jun 25;117(5):1295–308. doi: 10.1093/cvr/cvaa171 (PMC8064441; doi:10.1093/cvr/cvaa171)
Supplement: cvaa171_Supplementary_Data [file cvaa171_supplementary_data.zip › Suppl/CVR-2018-789R3 Online Supplemental Metformin.docx]

**Online Supplemental – Methods**

Metformin directly suppresses atherosclerosis in normoglycemic mice via haematopoietic Adenosine Monophosphate-Activated Protein Kinase (AMPK)

A. Seneviratne

L. Cave

G. Hyde

S.K. Moestrup

D. Carling

J.C. Mason

D.O. Haskard

J.J. Boyle

**Analysis of atherosclerosis.**

We analysed atherosclerosis in *Ldlr*-deficient mice by a minor adaptation of previous methods, detailed below. Animals were allocated to treatment groups to age-match and sex-match the treatment groups, and were analysed under code-blind conditions. The bone marrow recipients were all female. The first cohort were 1:1 male: female in both saccharin and metformin groups. We did not identify any significant sex differences. Mice gained weight similarly on metformin and control conditions. Bone marrow transplant did not significantly affect weight gain.

**General reagents.**

Oil Red O, dextrin, Sudan-IV, 4% buffered formal saline, Benzoin, hydrogen peroxide, bovine serum albumin, tetramethylbenzidine were all from Sigma-Aldrich, Poole, UK. All other reagents were from VWR, Lutterworth, Leicestershire, UK.

**Mice and diets**.

*Ldlr*-deficient mice (*Ldlr^-/-^*, homozygous *Ldlr^tm1Her^)* were maintained in-house and were originally from Charles River, a UK distributor for Jax. AMPK-knockout mice (*Prkab1*^-/-^) were from KOMP, maintained in-house and without obvious abnormal phenotype. Mice genotypes were determined by polymerase chain reaction.

Mice in the study were a 50:50 mix of male and female and were studied at the ages indicated. Animals were housed in a specific pathogen-free environment and studied according to UK Home Office regulations. Standard chow diet was used throughout.

**Bone marrow transplantation**

Mice at approximately 11 weeks age were transferred to irradiated food, bedding and Baytril broad spectrum antibiotic in acidified water. After 1 week, they subjected to 4Gy and 4Gy gamma-irradiation from a controlled shielded calibrated Cesium source. The irradiator was local to the CBS unit. The two doses were separated by 24h. Immediately after the second dose, donor mice were culled and bone marrow harvested by PBS flush from long bones. These were then injected into recipients at a ratio of 1 donor:1 recipient. The mice were maintained on antibiotic (Baytril), irradiated bedding and irradiated food for a further two weeks. Then they were transferred to standard chow and either metformin or saccharin.

Atherosclerotic lesions were immunostained for mouse PRKAB1 and terminal peripheral blood was genotyped by qPCR. The levels of *Prkab1* (the deleted gene, using primers specific for the deleted segment) and for *beta-Gal* (a marker gene inserted into the locus) were measured by qPCR and compared.

**Metformin treatment *in vivo***

Control drinking water was Milli-Q, with saccharin added to 1mg/mL. For metformin treatment, the control drinking water had metformin (Sigma-Aldrich) added, also to 1mg/mL. The drinking water was handled as aseptically as possible. Mice only had the saccharin or saccharin/metformin water to drink. The saccharin ensured steady drinking without dehydration due to unpalatability of metformin.

**Metabolic measurements.**

Lipoprotein, cholesterol and triglyceride analysis was using Randox clinical lipid measurement kits as before. Glucose was measured with a clinical glucose meter and disposable strips.

**Pathology**

**En face staining of aorta.**

Mice were killed by excess inhalation of carbon dioxide. Hearts and aortae were then flushed in situ with PBS by syringe calibrated via a cannula inserted in the left ventricle and an outlet created by incision of the right atrium, calibrated against against a pressure of ~110 cm water. After 10 min, the buffer was replaced with buffered 1% formal saline for 10 min, followed by a lipid staining solution containing 0.5% Sudan IV for a further 10 min. The heart and aorta were then removed and placed on ice cold PBS.

For each specimen, the aorta was cut at the arch start site. By using 85mm (0.025x 0.015 mm superfine tips) microdissecting scissors and 110mm Dumont, non-serrated dissection forceps, the extraneous fatty and connective tissue around the aorta were carefully trimmed off until a clear and transparent aorta was obtained. All small arteries were excised from each aorta specimen, and the remaining intact aorta was removed and transferred into a culture dish containing PBS. The entire aorta was then cut longitudinally from the heart near the innominate artery to the iliac bifurcation. The dissected aorta was destained with 80% ethanol for one minute to remove non-specific lipid staining, and was then washed in PBS and allowed to lay flat onto a Superfrost slide.

Quantification was performed by taking images with a macroscopic CCD camera, drawing around the en face aortic lesions and the entire aorta profile using Image J (NIH, USA). The lesion area fraction was calculated by dividing the mean lesion area by the mean area of the aorta and expressed as a percentage.

**Aortic root histology and quantification**. Cryosections of the aortic root were stained with Oil Red O and Mayer’s haematoxylin and analysed blind, as previously described ^1,2,3,4^. Immunohistochemistry Immunohistochemistry was performed by standard procedures on residual sections not required for analysis of lesion size. Primary antibodies, which were diluted as appropriate in PBS, were rat anti-CD68 (both from Serotec, Oxford, UK), alkaline phosphatase conjugated mouse anti-alpha actin (clone α1A4) (Sigma-Aldrich, Poole, UK). Primary antibodies were followed by biotinylated rabbit or goat anti-rat immunoglobulin (Ig) secondary (Dako) and ABC-peroxidase system (Dako) using 3,3'-diaminobenzidine tetrahydrochloride (DAB) as substrate. Results of immunocytochemistry are presented as a percentage area fraction of the aortic root or as the percentage of lesional cells, as analysed by Image J software above.

**Macrophage culture and stimulation**

Macrophage culture from humans and from mice were by minor modification of previous methods.

PBMCs were prepared by density-gradient centrifugation and monocytes purified by adhesion as before. Metformin (1’,1’-dimethylbiguanide, N,N-dimethylbiguanide) was purchased from Sigma-Aldrich (D150959-5G), dissolved aseptically in PBS to 10mM stock, and then diluted to 10μM final concentration in culture medium. Human aseptically prepared LDL was purchased from Calbiochem and oxidatively modified with hypochlorite 1mM as before ^5,6,7,8^.

Bones were collected aseptically from AMPK-KO or matched littermate control mice and flushed with PBS to collect bone marrow cells. The cells were then cultured in six T75 flasks in 10% FCS DMEM supplemented with antibiotics, L-Glu and 10% L929-conditioned medium (a source of M-CSF) for 7 days. Cells were then washed and adherent cells recovered by scraping into medium, counted and transferred to fresh 10% FCS DMEM in 24-well plates or 96-well plates. RT-qPCR Macrophages were lysed in a guanidinium-based buffer (RLT buffer, Qiagen, Manchester, UK) and RNA purified by silica-resin affinity (RNAEasy Mini, Qiagen) according to manufacturer’s instructions. Reverse transcription was performed with Invitrogen Superscript-II, following manufacturer’s instructions (Invitrogen, Life Sciences, Paisley, UK). Quantitative polymerase chain reaction (qPCR) on the same RNA samples after) was performed after using BioRad iCycler real time PCR, MesaGreen mastermix (Sybr Green/Taq, Eurogentec, Southampton, UK) and 100fmol.μL^-1^ custom primers (synthesized by MWG Biotechnology, Ebersberg, Germany). Primers were designed by a Primer3 algorithm accessed via the NCBI website (www.ncbi.nlm.gov). Additional validation was on a further five donors over a longer time course, using qPCR by the same methods. Primer sequences were as before.

**Gene knockdown**

siRNA was carried out by minor modification of previous methods. The si-RNA oligos were from Dharmacon and were pools of 4 sequences. Si-RNA was made into complexes with Interferin (PolyPlus, Strasbourg, France) by incubation of 100 pmol siRNA in 1μL with 1μL Interferin for 10 min, prior to addition to the cells in 1 mL medium with 10% serum for 24 hours, at 100pmol siRNA per 105 cells in each well. Where indicated, 10 μL AMS-liposomes were substituted for 1 μL Interferin and added to cells in IMDM without serum for 18 hours. In some experiments, the oligos were purchased from Qiagen instead of Dharmacon and these were targeted to the α-1 chain of AMPK (PRKAA1 gene) only and added at the same concentrations and with the same transfection protocol as the Dharmacon oligos.

**Microarray analysis was carried by a minor adaptation of previous methods**

Macrophages were lysed in guanidinium (RLT buffer, Qiagen, Crawley, UK) and RNA purified by silica-resin affinity (RNAEasy Mini, Qiagen, according to manufacturers 2 instruction). Sample quality was controlled with Nanodrop UV spectrophotometry and Bioanalyzer micro-LC. Samples were prepared for Agilent 4x44k and Affymetrix 1.0ST microarrays following respective manufacturer’s instructions. The service Oxford Genome Technology (OGT) was used for the array labelling, hybridisation and reading. The same RNA samples were used for both microarray platforms and both analyzed with GeneSpring GX12 Agilent. Initial validation was with quantitative polymerase chain reaction (qPCR) on the same RNA samples after reverse transcription (Invitrogen Superscript, manufacturers instructions) using BioRad iCycler real time PCR, MesaGreen mastermix (Sybr Green/Taq, Eurogentec, Southampton, UK) and 100fmol.μL -1 custom primers (synthesized by MWG Biotechnology, Ebersberg, Germany). Primers were designed by Primer3 (www.ncbi.nlm.gov). Additional validation was on a further five donors over a longer time course, using qPCR by the same methods. Bioinformatics Gene Ontology analysis was with GeneSpring and DAVID , and manual curation of GO-terms. Transcription factor (TF) binding site analysis was primarily with LASAGNA2.0 and PASTAA.

**Chromatin immunoprecipitation**

This followed a minor modification of previous methods ^5^. Macrophages were cultured at 10^5^ cells per well in 24-well plates. In the experiments shown, macrophages were treated with metformin 10μM for 4h. Cell biology grade reagents were used throughout. Supernatant was removed and cells were fixed with 200μL / well of 1% paraformaldehyde, for 15mins at 4°C, per well, then washed briefly in PBS and then fixation stopped with 200μL per well of 0.1M glycine in PBS, for 15mins at 4°C. The glycine was decanted and the plate frozen at -80°C until processing. The plate was thawed on ice, and wells extracted in 100μL / well nuclear lysis buffer (Active Motif Nuclear Lysis kit 40010, buffer AM-1, plus phosphatase inhibitor cocktail 1:20, plus PMSF 1:100, plus Protease inhibitor cocktail 1:100), with the aid of a silicone policeman. The lysates were then sonicated at 4°C for 10mins (Covaris sonicator, Model S220, Peak Power 105, Duration 600s, Duty Fcator 2.0, Cycles /Burst 200). This produced a 200bp band with THP-1 cells, indicating shearing to the correct size (oligonucleosomal). The lysates were then incubated with rabbit monoclonal anti-pATF1 (Abcam Ab7085, 50ng/mL) or rabbit IgG (Abcam monoclonal IgG non-binding control, Ab172730, 50ng/mL), at 4°C overnight rotating in 1.5mL Eppendorf-type tubes (VWR, non-siliconised, Mol Biol Grade) at 20rpm (Stuart rotator). Then Protein G Dynabeads (ThermoFisher) were added, 4μL per reaction, and rotated at room temperature for 2h. Neodymium magnets were used to collect the beads. The beads were next incubated in Proteinase K (Promega, Molecular Biology grade, DNA-ase-free, RNAse-free, 200μg/mL, in TBS with 0.1% SDS and 10mM CaCl_2,_ total volume 100μL/reaction) overnight at 65°C. These conditions generate selectivity for Proteinase K, which is more stable than most other proteases. Then, the digested beads were added to mixed Phenol:chloroform:isoamyl alcohol 25:24:1 (Sigma-Aldrich P3803), mixed thoroughly by vortexing and centrifuged (13000 x *g*, 10mins, 4°C). The top layer (100μL) was removed, and 400μL ethanol (molecular biology grade, Sigma-Aldrich or VWR) and 20μL 0.1M Na-Acetate added (ie in excess). The eppendorfs were then stored at -80°C for 48-72h to precipitate as much DNA as possible, then centrifuged at 18,000 x *g* for 3h. Most (>~90%) of the supernatant was removed taking care not to resuspend the pellet and the residual supernatant dried in a laminar flow cabinet overnight. Dried DNA pellets were checked and resuspended in 200μL molecular biology water (Sigma-Aldrich). They were stored at -20C and analysed by qPCR.

**ELISA**

ELISAs for LTB_4_, RvD_1_ and PGI_2_ were carried out on saved culture supernatants. Cayman Chemicals 96-well colorimetric ELISA kits were used, exactly according to manufacturer’s instructions (CAY520111, CAY500380, CAY50110). The same kit was used for both species (human and murine eicosanoids are chemically identical). Absorbance was measured at the manufacturer’s recommended wavelength, using a Tecan SpectraFluor 96-well plate reader.

**Phagocytosis assays**

Human MDM were cultured by adhesion-purification on BD Biosciences Falcon (354118) 8-well glass chamber slides at 10^4^ cells per well. They were cultured for 7 days in metformin or vehicle control (1:1000 PBS) in 10% AHS IMDM and then medium changed and phagocytic challenges added. These were 4µL/well fluorobrite beads 30 mins (Molecular Probes F13082), or apoptotic cells (ApC), OxLDL or IgG-coated beads. The apoptotic cells were RAW cells that were subjected to 120mins UV-irradiation and then left in PBS for 6h in a tissue culture hood, and then stained with Di-O [1:200 dilution of 1mg/mL Di-O in aseptic DMSO (3’3-dioctadecyloxacarbocyanine perchlorate (D4292, Sigma-Aldrich)]. Then the fluorescent-stained RAW cells were added to the hMDM at 1:1 ApC:hMDM i.e. 10^4^ ApC / well. LDL (Calbiochem 437644|) was purchased and diluted to 1mg/ml in PBS and then oxidised with 1mM Sodium hypochlorite (Sigma-Aldrich) for 24h. The LDL was then stained with Di-O [1:200 dilution of 1mg/mL Di-O in aseptic DMSO (3’3-dioctadecyloxacarbocyanine perchlorate (D4292, Sigma-Aldrich) the same as the RAW cells) centrifuged and resuspended in PBS and added to macrophages. The centrifugation and resuspension also generate adherent aggregates of LDL than are microscopically identifiable. Hypochlorite-adduction is a characterised method of LDL oxidation that mimics epitopes and adducts in atherosclerotic plaques and targets CD36^9,10,11,12,13,14,15,16,17,18^. It also obviates use of copper. The phagocytic stimuli were incubated with the macrophages for 30 mins and then supernatant decanted, and the cells fixed in 1% formaldehyde and counterstained with DAPI, and then imaged in 20% PBS / 80% Glycerol by a fluorescence microscope (Leica DM2500).

**Flow Cytometry**

Blood was drawn from the tail vein (~50µL) and then transferred into an eppendorf tube containing EDTA, mixed gently, and transferred into a 15mL Falcon tube with 5 volumes of RBC Lysis buffer [8.3g NH_4_Cl 1g NaHCO_3_ 1mL EDTA(100mM) 1L H_2_O Milli-Q (18MΩ osmotically purified water) (Ready to use after autoclave; stored at 4°C))]. Then the cells were incubated 4 minutes on ice and then 1 vol of PBS-BSA (0.5%) added. Cells were then centrifuged 10 minutes at 400g (rcf) at 4°C and the supernatant discarded. Lysis was redone if necessary. The supernatant was carefully aspirated, removing as much liquid as possible without disturbing the pellet. Next, the pellet was resuspended in 200 µl of PBS-BSA (0.5%) and transferred to round bottom 96 well plate, and centrifuge 320g (rcf) at 4°C for 5 minutes. The supernatant was discarded and cells resuspended in 10 µl PBS-BSA (0.5% [5 g BSA (Sigma; A9418-100G) 1L PBS 1X (Filtered and stored at 4°C)]) containing 1/50 (10µg/ml) 2.4G2 (2.4G2; BD Pharmingen #553145) and blocked for 10 minutes on ice.

Next, 10 µl of antibody mixture was added and stain for 15 minutes on ice Anti CD11b-PECy7 (1/100) Anti CD115-PE (1/100) Anti Ly6c-APC (1/100), and one of: Anti NK1.1-biot (1/100) Anti CD19-biot (1/100) Anti CD3-biot (1/100) in PBS-BSA (0.5%) CD16/CD32 purified CD11b-PECy7 (M1/70; BD Pharmingen #552850) Ly6c-biotin (AL-21; BD Pharmingen #557359) CD115-PE (AFS98; e-bioscience #12-1152-82) [NK1.1-APC (PK136; BD Pharmingen #550627] Streptavidin-Pacific Blue (Molecular probes #S11222). Isotype controls were: CD115 Rat IgG1 kappa Ly-6C Rat IgM kappa, CCR5 Rat IgG2a kappa, CCR2 Rat IgG2b kappa, CX3CR1 Mouse IgG2a.

Next, volume was made up to 200 µl with PBS-BSA (0.5%) and cells centrifuged 5 minutes at 320g (rcf) at 4°C. The supernatant was discarded and cells washed with 200µl PBS-BSA (0.5%). Next, 10 µl of Streptavidin-Pacific Blue (1/500) was added and stained for 20 minutes on ice. Complete to 200 µl with PBS BSA and centrifuge 5 minutes at 320g (rcf) at 4°C. Supernatant was discarded and wash with 200µl PBS BSA and then transfer to FACS tubes for analysis using a Beckman Coulter CyAN.

**Macrophage-induced VSMC apoptosis.**

This was by a minor adaptation of previous methods^19,20,21,22,23^. Human MDM were cultured for 7 days in vehicle or metformin, then 1:20 Di-O was added [1:200 of 1mg/mL 3’3-dioctadecyloxacarbocyanine perchlorate in aseptic DMSO (D4292, Sigma-Aldrich] for 30 mins and then medium was changed. VSMCs used were HCMED1-E6, a human coronary vascular muscle cell line with extended senescent lifespan. HCMED1-E6 were added at 10^3^ cells per well to macrophages at 10^4^ cells per well ^19,20,21^. After overnight culture, Propidium Iodide was added for 5mins, supernatant was decanted and cells fixed in 1% formaldehyde, counterstained in DAPI, and imaged by a fluorescence microscope (Leica DM2500). The co-incubation was carried out with 10µg/mL functionally-neutralising goat anti-IGF1 (R&D Systems ) or control goat IgG.

# Statistics

Data were graphed in stem-and-leaf plots and examined visually for distribution, and tested for normality using Shapiro-Wilk. Inherently paired data that were not significantly non-normally distributed were tested using Student’s paired t-test. Inherently non-paired data in two groups, that were not significantly non-normally distributed, were tested using Student’s t-test. Data that were not significantly non-normal, in multiple groups, were tested using One Way ANOVA and post-tested using Holm-Sidak correction for multiple simultaneous comparisons. Data that failed normality testing by Shapiro-Wilk, or that looked non-normal on visual examination, and fell in multiple groups, were tested using One Way ANOVA on Ranks (Kruskal-Wallis), with Dunn’s post-test. Unpaired data that were non-normally distributed were tested using Wilcoxon Rank Sum Test (Wilcoxon-Mann-Whitney).

**Supplemental Figure I Metformin suppresses lesional macrophage content**

A, Representative micrographs of CD68 / smooth muscle actin double immunofluorescence of aortic root lesions. Colours and treatment are as indicated on the image. Two images are shown for each treatment.

B-D, Quantification by 3 complementary methods. The treatment and control groups are as indicated. The Y-axes are respectively complementary morphometric variables. The counted number of macrophages in the lesions in each aortic root section is in B. The total area of CD68 immunofluorescence by planimetry is in C, and their ratio (which gives the average sizes of the macrophages) is shown in D. All 3 were significantly different with metformin treatment, p<0.05, Student’s t-test.

E, Images of whole aortae opened out *en face* from the first cohort. The lesions are too small and indistinct to be accurately visualised in a small photograph if the whole aorta is imaged. The metformin image includes a small residual deposit of iliac adventitial adipose (red).

F, Representative images of PRKAB1 immunofluorescence staining in aortic root sections. Colours as indicated, true colour, RGB camera. Scalebars as indicated. Reconstitution as indicated. Green arrowheads, valve leaflets and adventitia have PRKAB1-positive staining. Upper image, Reconstitution with *Prkab1*^+/+^ bone marrow leads to PRKAB1 staining in the lesion (Green) (white-edged filled green arrow). Lower image, reconstitution with *Prkab1*^-/-^ bone marrow leads to selective loss of PRKAB1 staining in the lesion (white open arrow) but not the normal leaflet or adventitial structures (green arrowheads).

G, Peripheral blood reconstitution with *Prkab1*^+/+^ or *Prkab1*^-/-^ marrow. Y-axis, difference between Prkab1 (deleted sequence) and Beta-Gal (insert) as Ct values, hence a logarithmic scale. Symbols, reconstitution and treatment as indicated. Each point, a different mouse. * significant difference between reconstitution with knockouts and WT littermates (p<0.05, ANOVA). NS, no difference between metformin-treated and saccharin.

**Supplemental Figure II Mouse total body mass on saccharin or metformin**

A, body weights in experiment 1, mice treated with saccharin-only or metformin+ saccharin in drinking water, from 10-25 weeks of age, then culled and analysed. Y-axis, body weight (g). X-axis, groups as indicated. NS, p>0.05 Student’s t-test, data passed normality.

A, body weights in experiment 2, mice subjected to bone marrow transplantation from *Prkab*^-/-^ or *Prkab1*^+/+^ marrow, then treated with saccharin-only or metformin+ saccharin in drinking water, then culled and analysed. Y-axis, body weight (g). X-axis, groups as indicated. NS, p>0.05, Kruskal-Wallis.

**Supplemental Figure III Summary of RT-qPCR data of selected metformin-modulated genes**

Overall schema of direction of modulation of genes by metformin, together with bundling by gene ontology (Rows). The columns are by genotype as indicated. Green up arrow, overall induction. Red down arrow, overall repression.

**Supplemental Figure IV Metformin induces classical murine M2 genes in an AMPK-(*Prkab1*)-dependent and *Atf1*-dependent manner**

Y-axes, fold induction by RT-qPCR and -2^ΔΔCt^ calculations. X-axes, genotypes. Gene names as indicated, following HGNC nomenclature. Each data point represents a different mouse, *p<0.05, n=8, one way ANOVA with Holm-Sidak correction for multiple comparisons, after normality testing (Shapiro-Wilk). Time-points were as indicated and were chosen from preliminary time-course data (dot shown).

**Supplemental Figure V Metformin increases anti-atherogenic eicosanoids PGI_2_ and RvD_1_ and suppresses pro-atherogenic eicosanoid LTB_4_ in an *Atf1*-dependent manner in human monocyte-derived macrophages.**

Eicosanoids are as indicated on each graph. Y-axes, calibrated levels of eicosanoid, measured as in Methods. X-axes, cell culture conditions (equivalent to qPCR in Figure 5). Each data point represents a different donor, n=6 *p<0.05, One way ANOVA with Holm-Sidak correction for multiple comparisons, after normality testing.

**Supplemental Figure VI Metformin increases anti-atherogenic eicosanoids PGI_2_ and RvD_1_ and suppresses pro-atherogenic eicosanoid LTB_4_ in an *Atf1*-dependent and AMPK (*Prkab1*)-dependent manner in human monocyte-derived macrophages.**

Eicosanoids are as indicated on each graph. Each data point is a different mouse. Y-axes, calibrated levels of eicosanoid measured as in Methods. X-axes, cell culture conditions (equivalent to qPCR elsewhere). Each data point represents a different mouse. *p<0.05, n=8, One way ANOVA with Holm-Sidak correction for multiple comparisons, after normality testing. Time-points were as indicated and were chosen from preliminary time-course data (dot shown).

**Supplemental Figure VII CD163 immunostaining.**

**A, Validation of CD163 immunostaining**

Splenic red pulp from a cohort mouse was imaged at x20 magnification. Isotype controls, channels, dual-labelling and merged channels were all as indicated. Yellow indicates Cd68+CD163+ splenic red pulp macrophages, as expected.

**B, CD163/CD68 colocalisation in metformin-stimulated atherosclerotic lesions.** CD68 (Alexa-488), CD163 (Alexa-568) and DAPI channels are as indicated, as is the merged channel. Yellow = colocalisation. Images representative of n=5 colocalisation experiments on n=5 mice. CD163 quantification on the entire cohort was on sections without second immunolabel.

**C, CD163/CD68 colocalisation in metformin-stimulated atherosclerotic lesions.** Example with full dual-reciprocal isotype controls. CD68 (Alexa-488), CD163 (Alexa-568), isotype controls and DAPI channels are as indicated, as is the merged channel. Images representative of n=3 experiments on n=3 mice. Images demonstrate that the immunostaining is specific.

**Supplemental Figure VIII Metformin induces HO-1 in an AMPK-dependent manner in mouse atherosclerotic lesions.**

Similar images obtained with metformin treatment at 1 week (not shown). No HO-1 was seen in saccharin-control lesions (not shown). Channels and treatments are shown (HO-1, Alexa 568, CD68-Alexa 488, DAPI). Yellow arrowhead = colocalisation. Images representative of entire cohort.

**Supplemental Figure IX Metformin does not detectably modify mouse circulating blood monocytes *in vivo***

A, Staining and gating strategy. FSC/SSC is used to set a live cell gate. Then the monocytes are gated positively on Cd115-positivity (expression of *Csf1r*) using PE dye. Then, monocytes are subclassified by Ly6c expression detected by APC dye. Then levels of each chemokine receptor are assessed (FITC).

B, quantification and statistics of subsets. The Y-axes are as indicated, n-numbers are as shown. None of the differences was significant (Student’s t-test p>0.05).

C, quantification and statistics of chemokine receptors. Expression levels were measured by mean fluorescence intensity and standard methods and n-values are as shown. There were no differences (p>0.05, Student’s t-test).

**Supplemental Figure X Metformin treatment of established disease increases lesional VSMCs and metformin promotes VSMC survival in macrophage co-cultures**

A, Graphical timeline of experiment.

B, Lesion planimetry based on Oil Red O analysis at the aortic root. N-values as shown, p<0.05, Student’s t-test. The numbers that were scored as having fibrous-cap-like structures are given.

C, representative micrograph of a fibrous cap-like structure in response to metformin treatment. Treatments as indicated. Colours are as indicated, representing anti-CD68-Alexa-488 and the α-smooth-muscle-actin-Cy3.

D, ELISA for IGF1 in the culture supernatants of hMDM (same experiments as in Figure 4). Y-axis, calculated IGF1 concentration, from calibrator. X-axis, metformin (10µM) treatment as indicated. (Data are mean ± SE, n=8, *p<0.05, t-test).

E, Validation of p-IGF1R immunostaining in formaldehyde-fixed cells. Bone marrow macrophages were cultured as before, fixed for 15 mins in 1% formaldehyde and then immunostained with phospho-IGF1R (Abcam), and a peroxidase/DAB secondary system. Isotype controls, phospho-IGF1R are as indicated. Control or insulin treatments are as indicated.

F, Representative micrographs of p-IGF1R in lesions. Treatment and staining as indicated (25-40 weeks). The additional subendothelial VSMCs are immunopositive for p-IGF1R. Inset, negative controls.

G, Macrophage-induced VSMC apoptosis, counted using DAPI and a macrophage-dye (methods) with hMDM and HCMED1E6 VSMCs, by a minor adaptation of previous methods^19,20,21^. X-axis, respective treatment, n=5 donors, Y-axis, VSMC-specific death. *p<0.05, Student’s t-test for indicated comparison. Anti-IGF1, goat anti-human neutralising antibody to IGF1 (R&D Systems), IgG – goat polyclonal IgG (R&D Systems).

**Supplemental Figure XI, Metformin and steatohepatitis**

A, representative photomicrographs, scalebars indicated lengths, colours and stains as indicated, ORO is a red lipid stain and hematoxylin is a blue nuclear counterstain.

B-D grading of different aspects of non-alcoholic steatohepatitis, according to a clinical grading scheme^24^. Y-axes, each score, X-axes, treatment, symbols as indicated.

References

1. Yun, S., Leung, V. W., Botto, M., Boyle, J. J., and Haskard, D. O.Brief report: accelerated atherosclerosis in low-density lipoprotein receptor-deficient mice lacking the membrane-bound complement regulator CD59. *Arterioscler.Thromb.Vasc.Biol.*; 2008;**28**:1714-1716.

2. Leung, V. W., Yun, S., Botto, M., Mason, J. C., Malik, T. H., Song, W., Paixao-Cavalcante, D., Pickering, M. C., Boyle, J. J., and Haskard, D. O.Decay-accelerating factor suppresses complement C3 activation and retards atherosclerosis in low-density lipoprotein receptor-deficient mice. *Am.J.Pathol.*; 2009;**175**:1757-1767.

3. Lewis, M. J., Malik, T. H., Ehrenstein, M. R., Boyle, J. J., Botto, M., and Haskard, D. O.Immunoglobulin M is required for protection against atherosclerosis in low-density lipoprotein receptor-deficient mice. *Circulation*; 4-8-2009;**120**:417-426.

4. Malik, T. H., Cortini, A., Carassiti, D., Boyle, J. J., Haskard, D. O., and Botto, M.The alternative pathway is critical for pathogenic complement activation in endotoxin- and diet-induced atherosclerosis in low-density lipoprotein receptor-deficient mice. *Circulation*; 9-11-2010;**122**:1948-1956.

5. Boyle, J. J., Johns, M., Kampfer, T., Nguyen, A. T., Game, L., Schaer, D. J., Mason, J. C., and Haskard, D. O.Activating transcription factor 1 directs Mhem atheroprotective macrophages through coordinated iron handling and foam cell protection. *Circ.Res.*; 6-1-2012;**110**:20-33.

6. Wan, X., Huo, Y., Johns, M., Piper, E., Mason, J. C., Carling, D., Haskard, D. O., and Boyle, J. J.5'-AMP-activated protein kinase-activating transcription factor 1 cascade modulates human monocyte-derived macrophages to atheroprotective functions in response to heme or metformin. *Arterioscler.Thromb.Vasc.Biol.*; 2013;**33**:2470-2480.

7. Boyle, J. J., Johns, M., Lo, J., Chiodini, A., Ambrose, N., Evans, P. C., Mason, J. C., and Haskard, D. O.Heme induces heme oxygenase 1 via Nrf2: role in the homeostatic macrophage response to intraplaque hemorrhage. *Arterioscler.Thromb.Vasc.Biol.*; 2011;**31**:2685-2691.

8. Boyle, J. J., Harrington, H. A., Piper, E., Elderfield, K., Stark, J., Landis, R. C., and Haskard, D. O.Coronary intraplaque hemorrhage evokes a novel atheroprotective macrophage phenotype. *Am.J.Pathol.*; 2009;**174**:1097-1108.

9. Westendorf, T., Graessler, J., and Kopprasch, S.Hypochlorite-oxidized low-density lipoprotein upregulates CD36 and PPARgamma mRNA expression and modulates SR-BI gene expression in murine macrophages. *Mol.Cell Biochem.*; 2005;**277**:143-152.

10. Marsche, G., Zimmermann, R., Horiuchi, S., Tandon, N. N., Sattler, W., and Malle, E.Class B scavenger receptors CD36 and SR-BI are receptors for hypochlorite-modified low density lipoprotein. *J.Biol.Chem.*; 28-11-2003;**278**:47562-47570.

11. Marsche, G., Levak-Frank, S., Quehenberger, O., Heller, R., Sattler, W., and Malle, E.Identification of the human analog of SR-BI and LOX-1 as receptors for hypochlorite-modified high density lipoprotein on human umbilical venous endothelial cells. *FASEB J.*; 2001;**15**:1095-1097.

12. Malle, E., Waeg, G., Schreiber, R., Grone, E. F., Sattler, W., and Grone, H. J.Immunohistochemical evidence for the myeloperoxidase/H2O2/halide system in human atherosclerotic lesions: colocalization of myeloperoxidase and hypochlorite-modified proteins. *Eur.J.Biochem.*; 2000;**267**:4495-4503.

13. Hazen, S. L., Hsu, F. F., Gaut, J. P., Crowley, J. R., and Heinecke, J. W.Modification of proteins and lipids by myeloperoxidase. *Methods Enzymol.*; 1999;**300**:88-105.

14. Assinger, A., Koller, F., Schmid, W., Zellner, M., Koller, E., and Volf, I.Hypochlorite-oxidized LDL induces intraplatelet ROS formation and surface exposure of CD40L--a prominent role of CD36. *Atherosclerosis*; 2010;**213**:129-134.

15. Hazell, L. J., Arnold, L., Flowers, D., Waeg, G., Malle, E., and Stocker, R.Presence of hypochlorite-modified proteins in human atherosclerotic lesions. *J.Clin.Invest*; 15-3-1996;**97**:1535-1544.

16. Malle, E., Hazell, L., Stocker, R., Sattler, W., Esterbauer, H., and Waeg, G.Immunologic detection and measurement of hypochlorite-modified LDL with specific monoclonal antibodies. *Arterioscler.Thromb.Vasc.Biol.*; 1995;**15**:982-989.

17. Hazell, L. J., van den Berg, J. J., and Stocker, R.Oxidation of low-density lipoprotein by hypochlorite causes aggregation that is mediated by modification of lysine residues rather than lipid oxidation. *Biochem.J.*; 15-8-1994;**302 ( Pt 1)**:297-304.

18. Hazell, L. J. and Stocker, R.Oxidation of low-density lipoprotein with hypochlorite causes transformation of the lipoprotein into a high-uptake form for macrophages. *Biochem.J.*; 15-2-1993;**290 ( Pt 1)**:165-172.

19. Boyle, J. J., Bowyer, D. E., Weissberg, P. L., and Bennett, M. R.Human blood-derived macrophages induce apoptosis in human plaque-derived vascular smooth muscle cells by Fas-ligand/Fas interactions. *Arterioscler.Thromb.Vasc.Biol.*; 2001;**21**:1402-1407.

20. Boyle, J. J., Weissberg, P. L., and Bennett, M. R.Human macrophage-induced vascular smooth muscle cell apoptosis requires NO enhancement of Fas/Fas-L interactions. *Arterioscler.Thromb.Vasc.Biol.*; 1-10-2002;**22**:1624-1630.

21. Boyle, J. J., Weissberg, P. L., and Bennett, M. R.Tumor necrosis factor-alpha promotes macrophage-induced vascular smooth muscle cell apoptosis by direct and autocrine mechanisms. *Arterioscler.Thromb.Vasc.Biol.*; 1-9-2003;**23**:1553-1558.

22. Boyle, J. J.Human macrophages kill human mesangial cells by Fas-L-induced apoptosis when triggered by antibody via CD16. *Clin.Exp.Immunol.*; 2004;**137**:529-537.

23. Aitman, T. J., Dong, R., Vyse, T. J., Norsworthy, P. J., Johnson, M. D., Smith, J., Mangion, J., Roberton-Lowe, C., Marshall, A. J., Petretto, E., Hodges, M. D., Bhangal, G., Patel, S. G., Sheehan-Rooney, K., Duda, M., Cook, P. R., Evans, D. J., Domin, J., Flint, J., Boyle, J. J., Pusey, C. D., and Cook, H. T.Copy number polymorphism in Fcgr3 predisposes to glomerulonephritis in rats and humans. *Nature*; 16-2-2006;**439**:851-855.

24. Kleiner, D. E., Brunt, E. M., Van, Natta M., Behling, C., Contos, M. J., Cummings, O. W., Ferrell, L. D., Liu, Y. C., Torbenson, M. S., Unalp-Arida, A., Yeh, M., McCullough, A. J., and Sanyal, A. J.Design and validation of a histological scoring system for nonalcoholic fatty liver disease. *Hepatology*; 2005;**41**:1313-1321.
